# Disparity in childhood stunting in India: Relative importance of community-level nutrition and sanitary practices

**DOI:** 10.1371/journal.pone.0238364

**Published:** 2020-09-01

**Authors:** Kajori Banerjee, Laxmi Kant Dwivedi

**Affiliations:** 1 International Institute for Population Sciences (IIPS), Mumbai, India; 2 Department of Mathematical Demography and Statistics, International Institute for Population Sciences (IIPS), Mumbai, India; BITS Pilani, INDIA

## Abstract

Despite rapid macro-economic growth, one-third of the global burden of childhood stunting is contributed by India. This burden is characterized by wide-spread geographical variation within the country. This paper explores two research questions: (i) are the drivers of severe and moderate stunting similar? (ii) differential endowments or policy-effect, how do community-level nutrition and sanitary practices affect inter-state differences? Using data from Indian National Family and Health Survey 4, 2015–16, six states holding different ranks in the stunting continuum are compared to Tamil Nadu, taken as the benchmark state due to its laudable performance in the health care sector. Applying quantile regression approaches, the difference in state-level performance is decomposed into detailed covariate effects (differential endowments) and coefficient effects (differential strength of association between the drivers and outcome). The explanatory variables are not similarly associated with severe and moderate stunting. Decomposition results demonstrate a significant role of community-level sanitation practices compared to child nutrition behaviour in explaining the inter-state disparity. Coefficient effects play a dominant role in the lower tail of HAZ distribution for the poor performing states indicating that the worse outcomes of these states are due to weaker policy effects of the control variables on stunting. Multi-sectoral approach, identification and differentiation between severe and moderate stunting cases can be more instrumental in managing and reducing the scourge. This paper also advocates the potential benefits of customizing centrally-launched policies as per the state’s performance and introducing the concept coproduction in the existing nutrition and health policy framework. This will instigate a feeling of ownership of the problem of childhood stunting among the policy consumers and strengthen the influence of policies on the outcomes.

## Introduction

**Fig 1:**
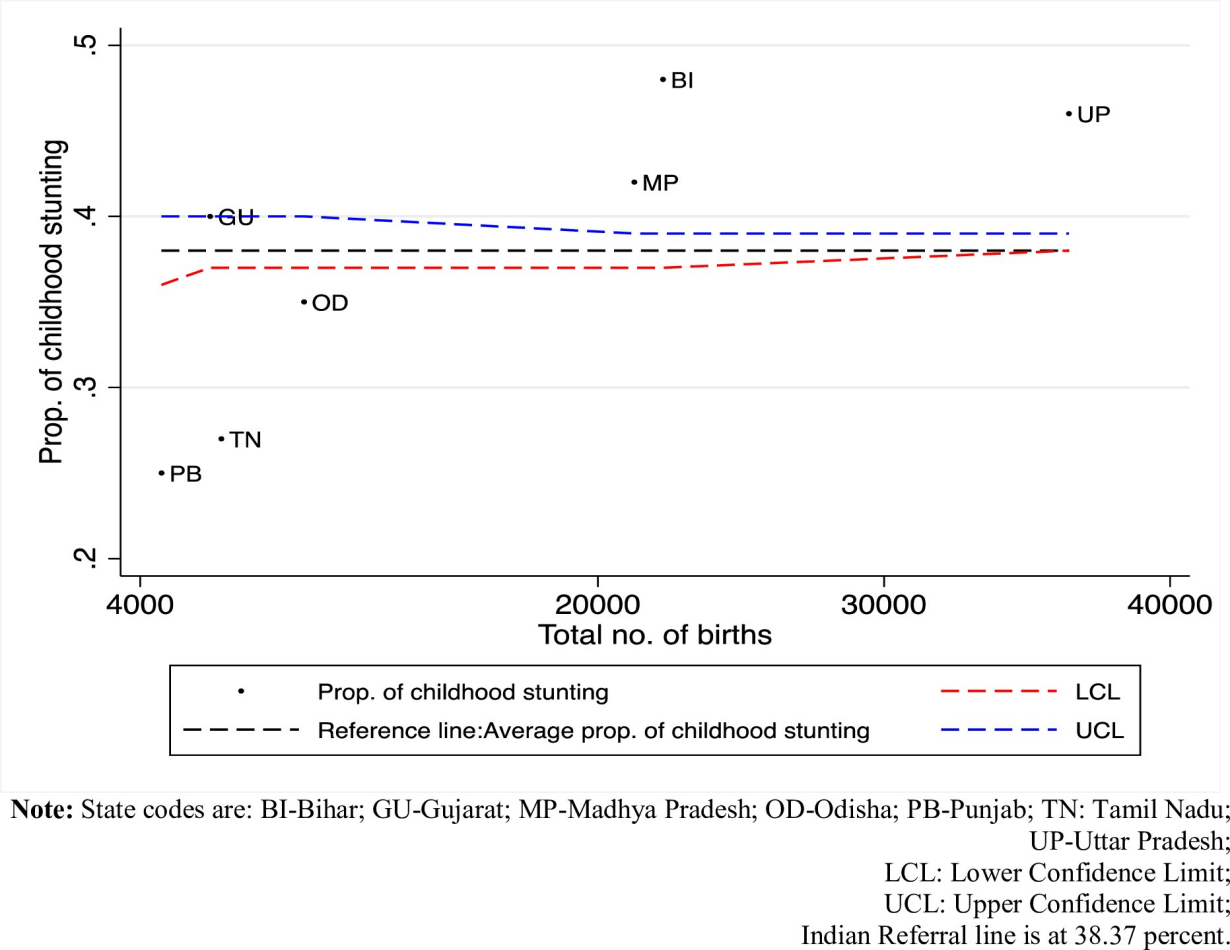
Funnel plot of percentage of stunting in total births, NFHS 4, 2015–16.

Stunting, low height for age (HAZ), has recently gained global attention and has been included as a target indicator in Sustainable Development Goal (SDG) 2; “zero hunger and end of all forms of malnourishment by 2030”. Out of 151 million stunted children in the world, one-third are found to reside in India; making the country an outlier even among the developing nations [1, 2]. The distinct feature of wide-spread geographical variations in child health outcomes in India is a major challenge in achieving SDG 2 [3–9]. The aggregate stunting in India has reduced by 10 percentage points over the last NFHS decade from 48% (2005–06) to 38% (2015–16) (S1 Fig). However, this decline is marked with extensive state-level heterogeneity. In 2015–16, Bihar, a Northern state in India, was pegged at the highest percentage of childhood stunting with 48% children under age five remaining stunted whereas the lowest was for Kerala, a demographically advanced Southern state in India, at 20%. The funnel plot in Fig 1 affirms the vast diversity in childhood stunting among the states of India. States such as Uttar Pradesh, Bihar, Madhya Pradesh are the upper outliers with very high fertility and percentage of childhood stunting whereas, states like Odisha, Punjab and Tamil Nadu are the lower outliers with lower fertility and percentage of childhood stunting. Studies have identified two main reasons for such cross-state disparities in child nutrition: “covariate effects” and “co-efficient effects”. Two states might differ in endowments, for example, differences in economic affluence, education, sanitary practices, feeding, and child care practices. This is captured by “covariate effects”. However, the differences in nutrition outcomes may also arise due to differences in strengths of association between the drivers and the nutrition outcome. This is expressed as “coefficient effects”. This can be interpreted as state-level variations in social and political commitments [10–13].

The persistently high levels of childhood stunting in states like Bihar, Uttar Pradesh, and Madhya Pradesh are a concern not only from a social, political, and public health standpoint but also because these states are projected to contribute the maximum to the future population growth in India [14]. On the other hand, Tamil Nadu is lauded for its superior performance in several public health fronts and often portrayed as the best practices state [15–19]. It has been successful in reducing childhood stunting level to 27% in 2015–16, which is comparable to many better performing middle-income countries of South-East Asia [2]. Along with being a good performer, Tamil Nadu also has a sizeable population, almost 6% of India’s population, which motivated us to take it as a comparison to the other six states.

Existing policies can be strengthened by exploring the potential drivers that fortified the performance of Tamil Nadu regarding childhood stunting. Several maternal, child and household characteristics are directly associated with childhood stunting. Previous literature documents a strong association of a child’s age, sex, and birth order with stunting [20–25]. Research also provides evidence that maternal educational attainment, anthropometric measures, social and economic conditions predispose a child’s stunting status [26–29]. Socio-economic conditions of the household play a major role in determining child health status as higher financial capabilities are linked with better care, health services, quality of food resulting in positive health outcomes [30, 31]. These characteristics are often found to be better in states like Tamil Nadu with impressive public health outcomes [17].

The novelty of the present study lies in evaluating the role of nutrition behaviour and sanitation practices in explaining the cross-state disparities. It has been well-established that nutrition behaviour in the form of child feeding practices and dietary diversity plays an essential role in determining childhood stunting status [18, 32–34]. However, recent studies have blamed unimproved sanitation facilities and preference of open defecation as one of the prominent environmental factors influencing the high prevalence of childhood stunting and negative child health outcomes in certain parts of India [35–44]. One-third of 2.4 billion people without proper sanitation facilities and two-third of 946 million people practicing open defecation worldwide are found in India [45]. Unhealthy sanitary practices and poor hygiene in the household and the community deteriorates the neighbourhood environment by infusing disease-carrying agents that cause enteric or intestinal diseases that affect the height of children more than mortality [42]. The problem is aggravated in densely populated countries such as India [36, 44, 46]. Evidence suggests that a large fraction of the difference between the heights of children in Sub-Saharan Africa and India can be explained by open defecation only [38]. Identifying the negative health implications of poor sanitary practices, the Government of India has strongly advocated for a reduction in open defecation through the “Swacch Bharat Mission” (Clean India Mission) and increased investment in the cause (from 411 million USD to 1297 million USD during 2014 to 2017 (1 USD = 69.43 INR)) [47].

In this paper, the potential contributors to regional disparities in childhood stunting has been bucketized under five groups: child variables, maternal variables, household socio-economic status, community nutrition behaviour, and community disease environment. Bihar, Uttar Pradesh, Madhya Pradesh, Gujarat, Odisha, and Punjab are compared to our selected bench-mark state of Tamil Nadu to assess the drivers of regional heterogeneity in India. The analysis uses the recent round of the National Family and Health Survey (NFHS) 4, 2015-16.This study applies a quantile regression (QR) approach where the full HAZ distribution is utilized. The differences between the states are decomposed into co-efficient and covariate effects using re-centered influence function quantile regression (RIF-QR) approach. The study aims to answer two research questions: (i) are the drivers of severe and moderate stunting similar? (ii) differential endowments or policies, how do community-level nutrition and sanitation interventions affect inter-state differences? Based on the findings of the study, we discuss some practical solutions for tackling the issue of childhood stunting and cross-state disparities to successfully achievethe health and hunger SDGs by 2030.

## Materials and methods

### Data

NFHS 4, 2015–16, a nationally representative cross-sectional two-stage stratified large scale sample survey, provides reliable estimates of maternal and child health in India [48]. The first round was conducted in 1992–93. All the rounds of NFHS are conducted in India under the stewardship of the Ministry of Health and Family Welfare with the International Institute for Population Sciences, Mumbai as the nodal agency.

The surveys provide anthropometric measures of children below 60 months. This study has the advantage of using the entire data set of height for age measures. To evaluate the change in HAZ distribution in the last NFHS decade, a detail of the sample size and HAZ distribution for NFHS 3, 2005–06, and NFHS 4, 2015–16 are provided in Panel 1 and 2, Table 1. The sample sizes of the seven selected states are provided in Panel 3, Table 1.

**Table 1:** Description of sample in the last two rounds of NFHS.

Description	NFHS 3 (2005–06)	NFHS 4 (2015–16)
**Panel 1: Sample description**		
Number of States in India from where sample was collected	28	29
Number of Union Territories in India from where sample was collected	1	7
	(National Capita Region: Delhi)	
Total number of households	1,09,041	6,01,509
Total number of eligible women (15–49 years)	1,24,385	6,99,686
Total number of mothers who gave birth in 5 years prior to the survey	36,850	1,90,898
Total number of children below 60 months	51,555	2,59,627
**Panel 2: Descriptive statistics of the dependent variable: Height for Age Z-Scores (HAZ) (excluding the children with missing height and age data)**		
Total number of children below 60 months taken for analysis excluding the ones with missing information in height or age	41306	225002
Mean	-1.87(-1.71)	-1.48(-1.48)
Standard deviation	1.66(1.67)	1.67(1.68)
Coefficient of variation	-0.89(-0.98)	-1.13(-1.13)
5th quantile	-4.52(-4.38)	-4.07(-4.08)
10th quantile	-3.92(-3.76)	-3.47(-3.48)
25th quantile	-2.94(-2.78)	-2.54(-2.54)
50th quantile: median	-1.93(-1.76)	-1.58(-1.58)
75th quantile	-0.87(-0.72)	-0.54(-0.54)
90th quantile	0.19(0.36)	0.57(0.58)
**Panel 3: Sample sizes of selected states from NFHS 4, 2015–16 (excluding the children with missing height and age data)**		
Bihar		22275
Uttar Pradesh		36465
Madhya Pradesh		21272
Gujarat		6444
Punjab		4746
Odisha		9728
Tamil Nadu		6836

The weighted descriptive statistics are provided for the dependent variable with the unweighted values within parentheses.

All union territories were excluded in NFHS 3. Telangana was awarded separate statehood in 2014. Hence, the data on Telangana is missing in NFHS 3. All other major states have had the same boundaries in the past decade.

The analysis excludes children with missing height and age data.

### Dependent variable

The continuous HAZ scores are taken as the dependent variable. Stunting is HAZ below minus 2 standard deviation units from the median of a reference population as devised by WHO. A child is considered to be severely stunted if the HAZ scores fall below minus 3 standard deviations [49–52]. Height for age is considered to be a measure of prolonged health degradation rather than inappropriate dietary intake in a transitory period [53]. Stunting is a measure of chronic undernutrition and a strong predictor of adult height. It has life-long degrading effects on cognitive abilities, physical impairments, and income [54–60]. The standing height of children older than 24 months was taken and the recumbent length of children below 24 months was measured using the SECA scale with a digital screen.

The mean of the HAZ distribution has increased from -1.87 to -1.48 over the last NFHS decade (Panel 2, Table 1). The standard deviations for both NFHS 3 and 4 are similar. The median of the distribution has improved from -1.93 to -1.58. The 90^th^ percentile cut-off has increased from 0.19 in NFHS 3 to 0.57 in NFHS 4. This indicates, 10% of children secured a HAZ score above 0.19 in 2005-06whichimproved to 0.57 in 2015–16. A positive shift in the later percentiles points at the improvement in overall HAZ scores. This shows that there has been a rightward shift in the distribution of HAZ. However, the shift for the lower quintiles, where the stunted children are concentrated has not been too impressive.

### Independent variables

Based on previous research we have listed out a few important child-, maternal-, household-, and community-level factors that are argued to be associated with childhood stunting. Table 2 provides the detailed distribution of the explanatory variables and the percentage of stunted children by the explanatory variables in NFHS 4.

- **Individual or Child variables:** Size of the child at birth, age of the child, sex, birth order of the child, and morbidity status within two weeks before the survey are included as demographic and health attributes of children.
- **Mother variables:** Mother’s age at birth, body mass index and education are included to define the demographic and social status of mothers.
- **Household socio-economic status:** Place of residence, religion, social class, wealth index is used to determine the household socio-economic status (SES).
- **Child nutrition status:** To capture the child nutrition performance in a state we used a modified version of the Child Nutrition Score (CNS) [61], deleting the stool disposal variable from the score as it was integrated with the disease environment variable. This score is based on nine child feeding practice measures ranging from 0 to 1. The items in the score are: (i) early initiation of breastfeeding, (ii) exclusive breastfeeding for children under age 6 months, (iii) timely introduction of complementary food to children aged 6–8 months, (iv) minimum dietary diversity for children aged 6–23 months, (v) minimum meal frequency for children aged 6–23 months, (vi) children aged 6–23 months consuming iron-rich food, (vii) children aged 6–59 months taking vitamin A supplements, (vii) proportion of children aged 6–59 months living in households using iodized salts and (ix) children aged 12–23 months who are fully immunized. The proportion of the items is taken and the score ranges from 0 to 9. It would be ideal if data on the effects of various nutrition policies were obtainable. However, due to limited data sources on such direct policy variables, this has been used as a manifested proxy to understand the policy commitment and achievements in the selected states. The indicator is estimated at the primary sampling unit level, capturing the community level nutrition behaviour.
- **Disease environment:** Unsafe stool disposal of child and community level practice of open defecation are taken as a proxy for the disease environment. Percentage of households in the primary sampling unit (PSU) with unimproved and no sanitation facilities are estimated.

**Table 2:** Percentage of childhood stunting by various explanatory variables.

Background characteristics	NFHS 4 (2015–16)	
	Percentage of stunted index children (below -2 s.d.)	Total children (proportion)
**Child variables (0–59 months)**		
**Size of child at birth**		
Average	37.83	155838 (0.69)
Large	34.29	38071 (0.17)
Small	46.85	26224 (0.12)
**Age of child**		
0–6 months	19.75	26726 (0.12)
6 months-1 year	25.86	18960 (0.08)
1–3 years	43.34	90615 (0.40)
3–5 years	41.47	88701 (0.39)
**Sex of child**		
Male	38.84	116360 (0.52)
Female	37.86	108642 (0.48)
**Birth order**		
1	37.71	166327 (0.74)
2	40.8	52353 (0.23)
3+	35.58	6322 (0.03)
**Had any morbidity in the last two weeks prior to survey**		
No	38.49	174702 (0.78)
Yes	37.95	50300 (0.22)
**Mother variables**		
**Mother's age at birth**		
Below 19years	41.56	15000 (0.07)
20–29 years	37.78	166427 (0.74)
above 30 years	39.58	43575 (0.19)
**Mother's Body mass index**		
Underweight	45.79	53285 (0.24)
Normal	38.08	139706 (0.62)
Overweight/obese	27.38	32007 (0.14)
**Education of mother**		
No education	50.76	68978 (0.31)
Primary	43.42	32835 (0.15)
Secondary	32.79	102191 (0.45)
Higher	20.81	20998 (0.09)
**Child Nutrition Score (CNS) for states**		
**CNS**		
Low	43.22	79805 (0.35)
Moderate	40.71	77569(0.34)
High	30.24	67628 (0.30)
**Disease environment**		
**Stool disposal**		
Safely disposed	31.21	88460 (0.39)
Not safely disposed	42.84	136472 (0.61)
**Place of defecation**		
Does not defecate in the open	31.42	130270 (0.58)
Openly defecates	47.20	94732 (0.42)
**Household characteristics**		
**Place of residence**		
Urban	30.99	53483 (0.24)
Rural	41.2	171519 (0.76)
**Religion**		
Hinduism	38.48	163089 (0.72)
Islam	39.8	35241 (0.16)
Others	29.75	17896 (0.08)
**Social Class**		
SC/ST	43.11	86980 (0.39)
OBC	38.7	88803 (0.39)
Others	30.92	40790 (0.18)
**Wealth Index**		
Poor	47.74	111492 (0.5)
Middle	36.5	45136 (0.20)
Rich	26.07	68374 (0.30)
**Total**	**38.37**	**2,25,002**

Children with no information on height and age are excluded

SC- Scheduled Caste, ST- Scheduled Tribe, OBC- Other Backward Class

### Analytical strategy

Quantile regression (QR) enables in identifying the covariates that influence severe and moderate stunting by dividing the distribution in various quantiles instead of a population-averaged score [62]. It allows for the explanatory variables to vary across the entire distribution of HAZ scores, especially the lower tail where the severely and moderately stunted children are clustered. This is unlike ordinary least square regressions which are modeled on the mean value of the dependent variable leading to loss of data. We base our discussion on the lower quantiles of the HAZ distribution to emphasize the association of the covariates with the stunting status of children.

Popular decomposition methods like Oaxaca-Blinder (OB) and its extensions only decompose the mean of a variable over the groups of interest [63–65]. Often, the difference is caused by the tail ends of the distribution where extreme malnourishment lies. Since we are more interested to estimate the effect of an independent variable on the HAZ differences in a population with varying characteristics between the states of India, the unconditional effects are preferred. Re-centered Influence Function Quantile Regression (RIF-QR) unconditional decomposition is used for the detailed decomposition into covariate and coefficient effects [13, 32]. The RIF regression facilitates decomposition to coefficient and covariate effects using the conventional OB procedure which has its advantages. First, the OB procedure helps in the decomposition of differences in two distributions to coefficient and covariate effects. Further, this can be decomposed into detailed contributions of individual variables [66].

The basic working algorithm behind a RIF decomposition is to first estimate a counterfactual distribution of child HAZ scores. It shows how the distribution of child HAZ would have been in state B if the covariates in state B had similar associations with HAZ as in state A. The “covariate effect” is obtained from the difference between the counterfactual and state A child HAZ scores distribution. The “coefficient effect” is derived from the difference between the counterfactual and state B child HAZ score distribution. The contribution of an individual variable to the aggregate covariate and coefficient effects is then calculated using the OB decomposition technique [64, 67]. The detail of the method is provided in S1 File. This decomposition exercise adds to the knowledge whether the states are differently endowed (covariate effects) or do they behave differently despite similar endowments (coefficient effects). The intercept and slope are allowed to vary over the entire HAZ distribution and the states supporting the estimation of the wide-spread regional heterogeneity, one of the prime challenges to attain SDGs in India.

## Results

### Inter-state heterogeneity in India

**Fig 2:**
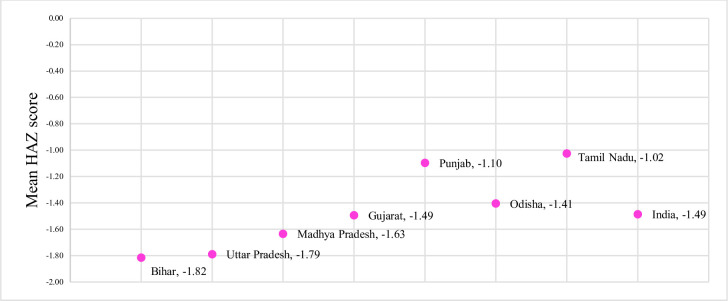
Mean HAZ in 2015–16 in India and selected states.

Fig 2 summarizes the mean HAZ scores of the selected states. The mean HAZ of Odisha and Gujarat was around the national average ranging between -1.41 to -1.49. Bihar, Uttar Pradesh, and Madhya Pradesh had the lowest mean HAZ. The highest mean HAZ is displayed by Tamil Nadu at -1.02 followed by Punjab at -1.10.

**Fig 3:**
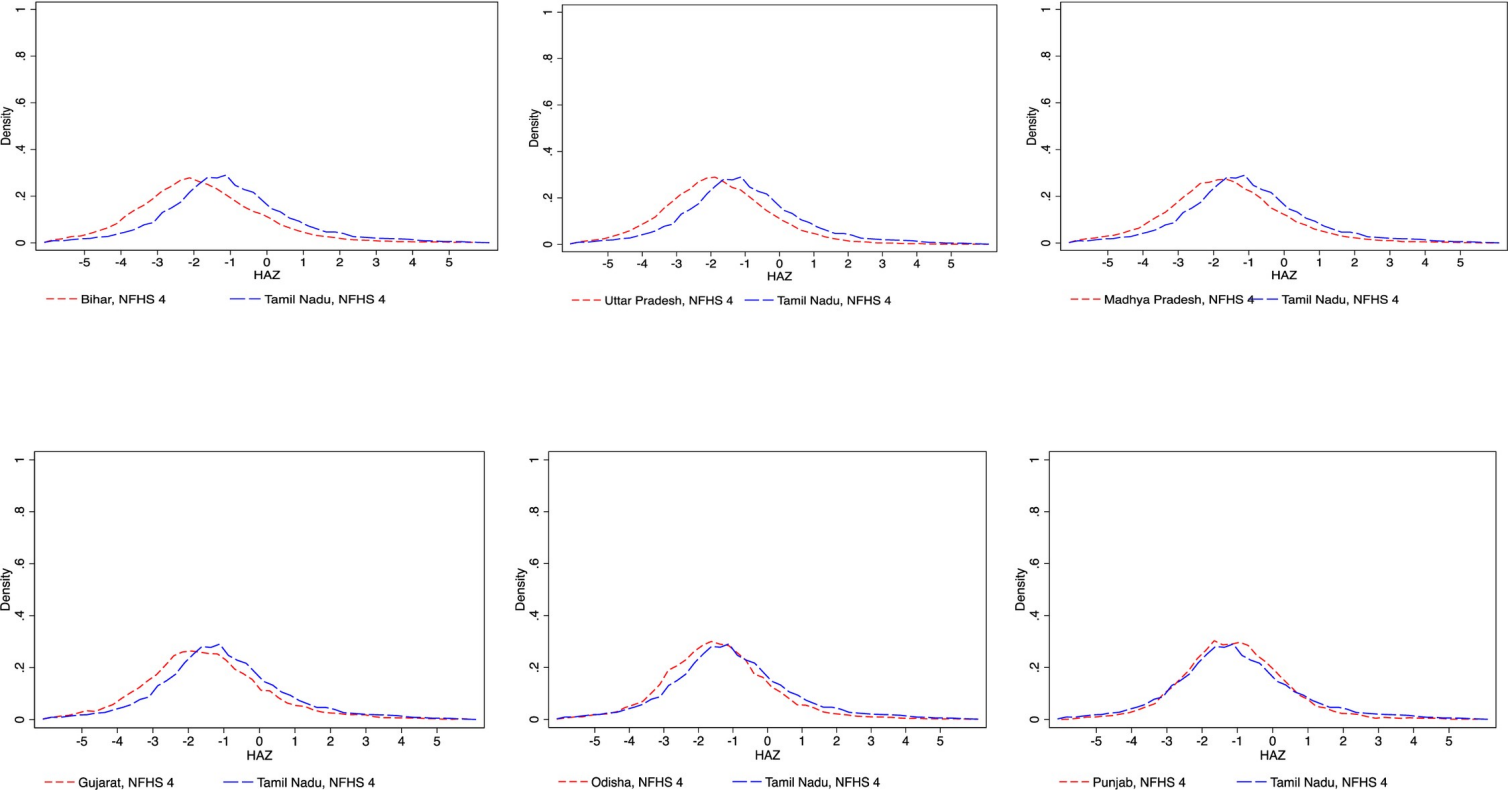
Comparing distribution of HAZ between states in India, 2015–16.

The distribution graphs in Fig 3 shows that Tamil Nadu is on the right side at the lower tail for all the states (except Punjab) indicating it to be a better performer. In the case of Bihar, Uttar Pradesh, and Madhya Pradesh the gap is found to be high in the lower tail. For Gujarat and Odisha, the gap with Tamil Nadu was high below the median. Punjab shows better performance in the lower tail of the HAZ distribution in comparison to Tamil Nadu.

The state-wise quantile regression results (S1–S7 Tables) highlight varying effects of the covariates on the different quantiles of the HAZ distributions of the states. For example, community-level child nutrition status does not have a significant effect on HAZ for most of the states. Its effect is significant in the lower quantiles of HAZ distribution only for Uttar Pradesh. However, either one or both the disease environment indicators; unsafe stool disposal, and percentage of households openly defecating in the community, are significantly associated with HAZ in most of the selected states.

Table 3 provides the results of the RIF decomposition for the inter-state differences in HAZ scores. The explained coefficient and covariate effects from the RIF decomposition of the selected states in comparison to Tamil Nadu are provided. The explained components are further decomposed by the contribution of each explanatory grouped variables.

**Table 3:** State-wise results of the RIF decomposition.

Selected States	10th Quintile	25th Quintile	50th Quintile	75th Quintile	90th Quintile
**Tamil Nadu versus Bihar**					
Difference	0.76*	0.82*	0.77*	0.75*	0.90*
Covariate (Endowments)	0.45*(58.97)	0.52*(62.97)	0.53*(69.01)	0.56*(74.46)	0.44*(49.05)
Coefficients	0.51*(66.91)	0.44*(53.48)	0.47*(61.00)	0.46*(61.08)	0.62 (69.18)
Interaction	-0.2*(-25.89)	-0.14*(-16.45)	-0.23*(-30.01)	-0.27*(-35.54)	-0.16 (-18.23)
**Covariate effect**					
Child variables	0.03*(6.99)	0.02*(4.37)	0.01 (2.73)	-0.01 (-1.06)	-0.03*(-6.18)
Mother variables	0.22*(49.51)	0.28*(54.61)	0.27*(50.37)	0.23*(40.68)	0.16*(37.1)
Nutritional status	0.01 (-0.77)	-0.01 (-1.24)	-0.01*(-1.21)	-0.01*(-1.63)	-0.02*(-3.98)
Sanitation	0.06*(13.77)	0.05*(9.41)	0.02 (4.01)	-0.01 (-2.15)	-0.06*(-13.21)
Household	0.14*(30.5)	0.17*(32.84)	0.24*(44.1)	0.36*(64.16)	0.38*(86.27)
**Coefficient effect**					
Child variables	0.76*(149.98)	0.52*(118.98)	0.45*(94.96)	0.36*(78.83)	-0.05 (-8.31)
Mother variables	0.03*(5.17)	-0.04*(-8.21)	-0.10*(-20.81)	-0.2*(-43.41)	-0.34 (-55.46)
Nutritional status	-0.1 (-20.28)	-0.03 (-6.91)	0.04 (8.3)	0.22 (47.31)	0.24 (39.06)
Sanitation	0.10* (19.26)	0.10* (21.71)	-0.07 (-14.18)	-0.34*(-74.99)	-0.19 (-31.38)
Household	0.23 (44.68)	0.09 (20.33)	-0.08 (-16.18)	-0.31*(-67.32)	-0.25 (-40.52)
**Tamil Nadu versus Uttar Pradesh**					
Difference	0.63*	0.71*	0.69*	0.75*	1.01*
Covariate (Endowments)	0.42*(67.75)	0.36*(51.01)	0.31*(45.45)	0.3*(40.33)	0.24*(24.15)
Coefficients	0.49*(77.53)	0.49*(68.17)	0.52*(74.59)	0.57*(76.04)	0.91 (90.34)
Interaction	-0.28*(-45.28)	-0.14*(-19.19)	-0.14*(-20.04)	-0.12*(-16.37)	-0.15 (-14.49)
**Covariate effect**					
Child variables	0.05*(11.8)	0.04*(10.11)	0.02*(5.67)	0 (0.19)	-0.02*(-7.7)
Mother variables	0.2*(47.45)	0.19*(51.09)	0.17*(53.63)	0.17*(54.98)	0.17*(69.98)
Nutritional status	0.03*(7.71)	0.01 (3.97)	0 (0.17)	0 (-0.86)	0.01*(3.66)
Sanitation	0.09*(20.06)	0.08*(21.46)	0.06*(20.42)	0.07*(23.83)	0.07*(30.47)
Household	0.06*(12.99)	0.05*(13.37)	0.06*(20.12)	0.07*(21.85)	0.01*(3.58)
**Coefficient effect**					
Child variables	0.87*(178.64)	0.49*(101.74)	0.37*(72.52)	0.3*(52.76)	0.12 (13.61)
Mother variables	-0.1 (-19.94)	-0.06 (-12.64)	-0.13 (-25.25)	-0.33*(-56.81)	-0.63 (-68.84)
Nutritional status	-0.31*(-63.59)	-0.20*(-40.8)	-0.07 (-13.27)	0.06 (11.17)	-0.09 (-9.99)
Sanitation	0.43*(88.65)	0.37*(75.4)	0.14 (27.19)	-0.03 (-4.51)	0.19 (20.97)
Household	0.12 (25.26)	-0.04 (-8.49)	-0.13 (-24.57)	-0.22*(-39.28)	-0.13 (-13.88)
**Tamil Nadu versus Madhya Pradesh**					
Difference	0.57*	0.61*	0.55*	0.58*	0.74*
Covariate (Endowments)	0.3*(53.27)	0.31*(50.52)	0.25*(45.15)	0.2*(34.99)	0.14*(19.5)
Coefficients	0.39*(67.51)	0.35*(58.44)	0.35*(63.49)	0.4*(68.36)	0.55 (73.79)
Interaction	-0.12 (-20.77)	-0.05 (-8.95)	-0.05 (-8.64)	-0.02 (-3.35)	0.05*(6.71)
**Covariate effect**					
Child variables	0.03*(8.52)	0.01 (4.39)	0 (0.18)	-0.02*(-10.95)	-0.03*(-22.5)
Mother variables	0.2*(65.19)	0.18*(59.79)	0.16*(63.48)	0.12*(59.39)	0.11*(73.61)
Nutritional status	0.01 (2.81)	0.01 (4.59)	0 (0.18)	0 (-0.08)	-0.04*(-30.35)
Sanitation	0.02 (6.72)	0.04*(11.56)	0.02 (8.09)	0.06*(28.63)	0.07*(45.77)
Household	0.05*(16.76)	0.06*(19.68)	0.07*(28.07)	0.05*(23)	0.05*(33.47)
**Coefficient effect**					
Child variables	0.60*(156.54)	0.42*(117.93)	0.36*(104.02)	0.25 (62.01)	0.26 (48.13)
Mother variables	-0.08 (-19.48)	-0.06 (-18.34)	-0.13 (-35.89)	-0.22*(-54.05)	-0.41 (-75.01)
Nutritional status	-0.22 (-56.52)	-0.24*(-66.7)	-0.07 (-20.88)	0.06 (13.87)	0.27 (49.15)
Sanitation	0.08* (22.02)	0.16* (46.15)	0.03 (7.66)	0.01 (2.51)	0.13 (24.54)
Household	0.1 (27.19)	-0.04 (-12.13)	-0.13*(-36.28)	-0.24*(-60.11)	-0.28 (-50.88)
**Tamil Nadu versus Gujarat**					
Difference	0.47*	0.49*	0.44*	0.44*	0.54*
Covariate (Endowments)	0.22*(46.12)	0.2*(40.41)	0.17*(38.47)	0.16*(37.63)	0.2*(37.08)
Coefficients	0.4*(84.31)	0.39*(79.21)	0.37*(84.77)	0.4*(91.15)	0.47 (87.35)
Interaction	-0.14*(-30.43)	-0.1*(-19.62)	-0.1*(-23.23)	-0.13*(-28.77)	-0.13 (-24.43)
**Covariate effect**					
Child variables	0.06*(28.33)	0.04*(19.06)	0.01 (5.96)	0.01 (3.97)	-0.01*(-3.7)
Mother variables	0.13*(57.74)	0.10*(48.26)	0.10*(57.42)	0.08*(47.59)	0.1*(51.1)
Nutritional status	-0.03 (-15.11)	0.01 (0.79)	0.01 (-2.56)	0.01 (7.49)	0.03*(16.01)
Sanitation	0.01 (3.07)	0.01 (-2.11)	-0.01 (-5.61)	0.01 (5.85)	0.04*(21.85)
Household	0.06*(25.96)	0.07*(34)	0.08*(44.8)	0.06*(35.1)	0.03*(14.74)
**Coefficient effect**					
Child variables	0.92*(231.33)	0.61*(157.99)	0.42*(113.89)	0.6*(151.21)	0.48 (101.3)
Mother variables	-0.03 (-6.28)	-0.04 (-11.43)	-0.03 (-9.22)	-0.17 (-42.66)	-0.34 (-73.31)
Nutritional status	0.06 (14.86)	-0.14 (-36.71)	-0.04 (-10.96)	-0.03 (-6.66)	-0.25 (-54.34)
Sanitation	-0.08 (-19.81)	-0.01 (-1.31)	-0.11 (-28.83)	-0.27*(-67.53)	-0.21 (-45.38)
Household	0.16 (40.2)	0.16 (41)	-0.11 (-31)	-0.18 (-45.49)	-0.26 (-55.49)
**Tamil Nadu versus Punjab**					
Difference	-0.24*	-0.07*	-0.02	0.14*	0.54*
Covariate (Endowments)	-0.43*(176.66)	-0.25*(361.57)	-0.18*(1090.16)	-0.18*(-134.99)	-0.31*(-57.52)
Coefficients	-0.03 (11.26)	0.06 (-90.35)	0.13*(-786.78)	0.35*(259.65)	0.9 (168.37)
Interaction	0.21 (-87.92)	0.12 (-171.22)	0.03 (-203.38)	-0.03 (-24.66)	-0.06 (-10.85)
**Covariate effect**					
Child variables	0 (-0.21)	0 (1.59)	-0.01 (2.83)	-0.01 (4.05)	-0.01*(3.08)
Mother variables	0.01 (-1.17)	0.01 (-5.73)	0.04*(-20.55)	0.03*(-18.12)	0.05*(-15.33)
Nutritional status	0.01 (-2.58)	0.01 (-3.33)	0 (-0.17)	0 (-1.69)	0.01*(-2.04)
Sanitation	-0.16 (37.22)	-0.06 (24.11)	-0.03 (18.63)	-0.06 (31.41)	-0.14 (45.13)
Household	-0.29*(66.73)	-0.21*(83.36)	-0.18*(99.27)	-0.15*(84.36)	-0.21*(69.16)
**Coefficient effect**					
Child variables	0.67*(-2442.48)	0.24 (375.91)	0.12 (93.01)	-0.11 (-30.69)	-0.48 (-52.64)
Mother variables	-0.12 (432.59)	-0.06 (-100.89)	-0.14 (-104.5)	-0.33*(-92.51)	-0.27 (-29.74)
Nutritional status	-0.28 (1008.98)	-0.23 (-357.4)	-0.08 (-57.52)	0.02 (6.78)	-0.12 (-12.95)
Sanitation	0.12 (-423.44)	0.04 (65.77)	0 (3.74)	-0.04 (-10.25)	0.05*(5.35)
Household	-0.35 (1282.83)	-0.16 (-257.37)	-0.17 (-129.04)	-0.15 (-42.74)	0.3 (33.17)
**Tamil Nadu versus Odisha**					
Difference	0.13*	0.31*	0.32*	0.45*	0.71*
Covariate (Endowments)	0.33*(260.34)	0.37*(121.74)	0.34*(107.73)	0.4*(88.13)	0.36*(51)
Coefficients	-0.07 (-55.23)	0.02 (7.01)	0.08*(25.86)	0.23*(51.1)	0.44 (63.01)
Interaction	-0.13*(-105.1)	-0.09*(-28.75)	-0.11*(-33.58)	-0.18*(-39.24)	-0.1*(-14.01)
**Covariate effect**					
Child variables	0.01 (1.91)	0.02*(4.05)	0.01 (3.45)	0.01 (2.12)	-0.01*(-3.06)
Mother variables	0.19*(58.49)	0.2*(52.51)	0.14*(41.16)	0.16*(40.21)	0.15*(41.51)
Nutritional status	0.01 (0.49)	0.01 (-0.35)	0.01 (-0.36)	0.01 (-0.48)	0.01*(-0.62)
Sanitation	0.07*(20.96)	0.04* (10.08)	0.01 (3.64)	0.04 (9.41)	0.01*(2.19)
Household	0.06 (18.16)	0.13*(33.72)	0.18*(52.11)	0.19*(48.74)	0.22*(59.99)
**Coefficient effect**					
Child variables	0.22 (-317.78)	0.21 (1002.04)	0.22*(267.58)	0.09 (38.91)	0.11 (23.64)
Mother variables	-0.03 (46.25)	-0.18 (-857.2)	-0.13 (-160.76)	-0.36*(-156.35)	-0.56 (-125.44)
Nutritional status	-0.3 (427.83)	-0.05 (-227.94)	0.02 (21.54)	0.21 (90.04)	0.12 (26.41)
Sanitation	0.19 (-265.35)	0.07 (346.56)	-0.09 (-113.2)	-0.19 (-84.14)	-0.12 (-25.99)
Household	0.06 (-91.86)	-0.05 (-229.13)	-0.15*(-177.06)	-0.27*(-116.76)	-0.33 (-75.23)

* indicates values are significant at p-value<0.005

Within parentheses total covariate and coefficient effects are given as percentages of the total difference explained.

Explanatory variable wise: Within parentheses the percentage explained out of total covariate/ coefficient effect is given.

For the selected states, except Punjab and Odisha, coefficient effects play a higher and significant role in explaining the differences in the HAZ distribution in the lower quantiles. For Tamil Nadu versus Bihar, the coefficient effects contribute 53 to 66% below the median. Maternal and child coefficient effects along with sanitation practices contributed significantly to explaining the differences below the median of the distribution. In case of Bihar, covariate effects were also important at the 25^th^ and 50^th^ quintiles, explaining 62 to 69% of the HAZ gap with Tamil Nadu.

In the case of Tamil Nadu versus Uttar Pradesh coefficient effects explained above 68% of the differences below the median. The largest significant contributors to coefficient effects are child variables, and community-level nutrition practices and sanitation behaviour.

For Madhya Pradesh versus Tamil Nadu, coefficient effects explain 58 to 67% of the differences below the median. Child coefficient effects and sanitation practices below the median, nutrition behaviour (at 25^th^quantile), and maternal variables (at the median) are found to be significant.

The coefficient effect contributes 79 to 85% to the differences (below median) between Tamil Nadu and Gujarat. Among the coefficient effect, child variables below the median contribute significantly.

Although in most of the poorer performing states covariate effects were small but that does not render them negligible. For the lowest quintile where the most severely stunted children are clustered, Tamil Nadu’s superior endowments of maternal health, education, community-level sanitation practices and household socio-economic status explains up to 35% of the HAZ gap with Bihar, Uttar Pradesh, Madhya Pradesh and Gujarat. In Uttar Pradesh, community-level nutrition practices contributed to 5% of the HAZ gap with Tamil Nadu.

For Punjab versus Tamil Nadu, Punjab displayed higher HAZ scores up to the 50^th^ quantile. Covariate effects are to be credited for this difference. The household SES endowments contribute 66 to 100% between the 10^th^ and 50^th^ quantiles.

In the case of Odisha, covariate effects are more dominating in explaining the differences at all quantiles. The disease environment contributes 21% to the covariate effects in the lowest quantile. Maternal endowments and household SES contributed more than 80% to the covariate effects in the lower quintiles.

## Discussion

India has been criticized for its slow progress in tackling childhood stunting despite its rapid macroeconomic growth. While many studies have attempted to identify the correlates of childhood stunting, very few have explored the association of covariates over the entire distribution of stunting and addressed the wide gaps in progress between the states of India. We identified a dearth of studies that: (i) discuss differential policy approaches to address moderate and severe stunting, and (ii) explore factors that explain the gaping regional heterogeneity in childhood stunting in India. A growing body of literature has blamed unhygienic sanitary practices rather than a lack in efforts to improve the quality of nutrition intake [35, 38, 68] for the unsatisfactory improvement in childhood stunting in India. Adding to the extant literature, the present study explores the relative importance of community-level disease environment and nutrition practices in affecting childhood stunting. Our analysis has three main insights pertinent from a policy standpoint.

First, our results are consistent with previous literature and confirm that the risk factors of severe and moderate stunting are not constant throughout India [69]. States with a higher prevalence of severe stunting should target the factors that have a stronger association with severe stunting. For example, in case of Uttar Pradesh, birth order of child, maternal education, community-level nutrition practices, unhygienic sanitary practices in the community like unsafe stool disposal and lack of improved sanitation facilities in the households, household wealth status has a stronger and significant effect in the lower quantiles of the HAZ distribution. However, for Tamil Nadu, community-level nutrition practices and sanitary habits did not have any significant effect at the lower quantiles. In Tamil Nadu, individual-level factors rather than community-level factors contribute to lowering the HAZ scores. This implies that targeting similar variables in different states of India without considering their present stunting levels can be ineffective. In case of Uttar Pradesh, monitoring nutrition and health requirements of higher parity births, strengthening universal coverage of the health facilities to monitor growth failures in children irrespective of the wealth status of the household, improving community-level education on age-appropriate nutrition practices for children, and promoting hygienic sanitary practices at the community-level can significantly improve HAZ scores. In case of Tamil Nadu, monitoring growth failures in children between the age of 1–3 years, improving parental education and awareness, and discouraging unsafe disposal of stool of children can effectively contribute to improvement of HAZ scores at the lower quantiles. Community involvement and holistic development has to be integrated in policy approaches to bolster the HAZ scores in Uttar Pradesh whereas policies emphasizing individual awareness can strengthen HAZ outcomes in Tamil Nadu.

Secondly, for most of the worse performing states like Bihar, Uttar Pradesh, Madhya Pradesh and Gujarat, coefficient effects explained the higher stunting rates as compared to Tamil Nadu. This indicates that policies concerning childhood stunting are not optimally effective in these states. This has inhibited these states to perform their best despite development-oriented leadership, policy and political permanency, and robust economic growth [70]. Weak association of community-level sanitation practices with stunting outcomes contributed majorly in explaining the worse performance of these states in comparison to Tamil Nadu. Studies assessing drivers of inter-regional HAZ differences advocate that child feeding practices and nutrition behavior directly contribute to improving the performance of one region against another in developing countries [18, 19, 32]. However, at the community level, the disease environment manifested through sanitation practices, is a vital driver in elucidating inter-state differences. Community-level nutrition practices were a significant driver in explaining the gap only in the case of Uttar Pradesh. This indicates that policies and programmes for both sanitation and nutrition improvement at the community level are crucial for Uttar Pradesh. The findings support that a disease-free environment coupled with proper energy and nutrient intake can facilitate optimal child growth and reduce the prevalence of childhood stunting significantly. The insignificant impact of nutrition and feeding behaviour might be an indication of weak policy effects of sanitation campaigns in the poor performing states of India. The intersection of Ministries in charge of nutrition and sanitation interventions and improving their shared knowledge base and coordination can effectively bolster the child nutrition outcomes in the poor performing states [71].

Thirdly, in case of the better performing states like Punjab and Odisha, covariate effects are more significant in explaining their HAZ differences with Tamil Nadu. Both Punjab and Odisha have shown remarkable progress in terms of child health and this is mainly due to an increase in the proportion of the population with better maternal health, education, and household wealth status. Nutrition policies and programmes in Odisha have improved since the 2000s due to the parallel and synergized functioning of several departments [72]. Odisha has adopted various innovative political and social approaches by collaborating Departments of Health and Family Welfare and Women and Child Development [73]. In addition to this, we suggest the involvement of the Ministry of Drinking Water and Sanitation to generate evidence on nutrition outcomes and sanitation facilities. Results from decomposition imply that sanitation practices along with improvement in maternal endowments and household SES can assist in further improvement in Odisha. A study suggests, Odisha could avert a substantial number of stunting cases by 2030 if the state scaled-up their sanitation practices to the standards of Tamil Nadu [18]. Post green revolution in the 1960s, Punjab has witnessed rapid agricultural growth along with an increase in per capita income and lower levels of calorie-related undernourishment [74]. Our findings indicate that Punjab is a positive deviant mainly due to the better socio-economic endowments of the state. Evidence support that Uttar Pradesh, Gujarat, Odisha were among the states with a high relative deprivation for stunting in the poorest wealth group whereas Tamil Nadu and Punjab were among the states with the lowest relative deprivation [70]. The main goal of public policies and programmes is to provide goods and services at a subsidized rate to the underprivileged sections of society. If public programmes function efficiently then the influence of the household’s socio-economic status and other maternal and child health indicators on childhood stunting should be not be statistically significant. This is because the low-cost goods and services provided to the poorer sections of the society will have a greater influence on developing their conditions than their purchasing power [30]. In states where performance has improved due to better SES of households, like Punjab, one should be cautious from a policy standpoint as the focus is to promote equitable policies that can reduce socio-economic inequality in child undernourishment. In conclusion, the paper suggests some policy reformations that might strengthen the effects of existing programmes targeted to reduce childhood stunting in India.

## Conclusion

Macro-economic growth may be a necessary but not a sufficient condition for improving child nutrition. For example, despite the slow economic progress of Brazil, utilization of radical social policies encouraged market-oriented reforms such as synergetic systems involving the poor and underprivileged population in the mechanism of growth and construction of national, nutritional and social policies and resulted in a significant reduction in poverty and under-nutrition [75]. Our results highlight that there is a weak association between policy and childhood stunting outcomes in the poor performing states compared to Tamil Nadu. The wider social and political mechanism through which sanitation can be improved to better nutrition outcomes has been less traversed [76]. In this light, we discuss the potential benefits of customizing centrally-launched policies and introducing the concept of co-production in the nutrition and health policy framework of India, especially in the poor performing states.

Tamil Nadu has customized various centrally launched schemes to accelerated improvement in childhood malnourishment scenario. For example, Tamil Nadu Integrated Nutrition Programme (TNIP), which was merged with the Integrated Child Development Scheme (ICDS) in 1990, has a two-worker model unlike the one-worker model followed by most of the other states in India. In TNIP-ICDS, one worker focusses on service provision for severely malnourished children whereas another staff member addresses malnourishment in older children [16]. Another customized scheme in Tamil Nadu is the universal Public Distribution System (PDS). In 1997, PDS was converted into a subsidy scheme targeting only population below the designated poverty line. The prices of ration was lower for the households belonging to below poverty line compared to those above the poverty line. The conceptual and operational issue of the dual price system of PDS is its inability to identify the actual resource poor households. However, Tamil Nadu continued PDS as a universal coverage scheme which ensured inclusion of several needy households which were not “officially” poor [77]. In the two better performing states in this study, Tamil Nadu and Punjab, PDS has a low exclusion rate of BPL families (less than 20%). Many poor performing states can take these examples and customize existing nutrition policies to enhance their efficiency in tackling stunting and malnutrition.

In context to the ongoing coronavirus pandemic, there has been a shift in Governance priorities towards safeguarding macroeconomic metrics in most countries, including India. There has also been unprecedented demands from the health sector during this crisis. However, programmes and health facilities monitoring growth failures cannot be neglected primarily because children with growth faltering also have lower immunity and can stand at risk of being infected at such a time of crisis. Thus, simultaneous efforts are required to shield stunted children from infection and thwart the long-term effects of childhood growth failures. Along with the improvement in management of the coronavirus infection in the country and amelioration of the impact of the pandemic, we advocate introduction of the concept of coproduction in nutrition-related interventions. Coproduction is the process through which citizens can take an active part in framing the policies and programmes by “producing public goods and services that are of consequence to them” [78]. “Institutionalized coproduction” [79, 80] in the public health sector can ensure active interaction between public health workers and policy advisors with the clients that can lead to increase in policy effectiveness through mutual understanding of the ground reality, harboring trust between the service providers and consumers and collection of true and useful information that can be valuable inputs in policy framing. The central idea is to make the people feel responsible and vested towards reducing childhood stunting by focussing on the determinants. In this paper, community-level sanitation behaviour emerges as one of the important drivers of inter-state disparity. Tamil Nadu has taken remarkable measures to ensure a rapid reduction in open defecation through the process of coproduction. Public Affairs Centre (PAC), located in Bengaluru, conducted a sanitation project in six districts of Tamil Nadu (Dharmapuri, Kanyakumari, Krishnagiri, Perambalur, Tirunelveli, and Tiruchirappalli) based on the idea of “Joint Action Committees” involving the service providers and beneficiaries to monitor and evaluate progress and fallouts of the Clean India Movement or “Swacch Bharat Abhigyan”. During this project, the PAC identified the dominance of women in the Village Poverty Reduction Committee, Panchayat Level Federation, and Self-Help Groups in these districts and employed them as “sanitation ambassadors” or “swacchtadoots”. In some districts of Tamil Nadu, like Salem, women were trained in masonry to become “rani mistris” to build toilets. Thus, women in Tamil Nadu became powerful agents of change in encouraging communities to accept and adopt safe sanitary practices. Many schools in Tamil Nadu enrolled students in the “sanitation club” which promoted innovative ideas to address the issue of sanitation among adolescents. Several such social and political reforms in Tamil Nadu offer a perfect example of how coproduction can be instrumental in improving stunting outcomes by directly affecting the determinants. The neighbouring country of Bangladesh has also reaped the benefits of coproduction in the case of sanitation. The Government of Bangladesh has adopted Community-led Total Sanitation (CLTS) programme to instigate social mobilization regarding open defecation. This approach uses participatory rural appraisal (PRP) where the community members themselves, evaluate their sanitation status concerning open defecation and extent of fecal-oral contamination [81]. This has helped in developing a feeling of ownership, shame, and disgust among the community members, which in turn has made the National Sanitation Programme of Bangladesh a classic success story. There is no “silver bullet” policy to address stunting uniformly. The findings highlight that identification and differentiation between severe and moderate stunting cases can be more instrumental in managing and reducing the scourge. This paper also advocates integrating nutrition and sanitation interventions at the community level and instigating a feeling of ownership of the problem of childhood stunting among the policy consumers in India to strengthen the influence of policies on the outcomes.

## Supporting information

S1 Fig Childhood stunting scenario in India, 1992–2016. (DOCX)

S1 Table Quantile regressions for Bihar, 2015–16. (DOCX)

S2 Table Quantile regressions for Uttar Pradesh, 2015–16. (DOCX)

S3 Table Quantile regressions for Madhya Pradesh, 2015–16. (DOCX)

S4 Table Quantile regressions for Gujarat, 2015–16. (DOCX)

S5 Table Quantile regressions for Punjab, 2015–16. (DOCX)

S6 Table Quantile regressions for Odisha, 2015–16. (DOCX)

S7 Table Quantile regressions for Tamil Nadu, 2015–16. (DOCX)

S1 File Method description. (DOCX)
