# Supplementary material for: Disparity in childhood stunting in India: Relative importance of community-level nutrition and sanitary practices
Source: PLoS One. 2020 Sep 1;15(9):e0238364. doi: 10.1371/journal.pone.0238364 (PMC7462311; doi:10.1371/journal.pone.0238364)
Supplement: S1 Fig — (DOCX) [file pone.0238364.s001.docx]

**Figure S1. Childhood stunting scenario in India, 1992-2016**
