# Supplementary material for: Disparity in childhood stunting in India: Relative importance of community-level nutrition and sanitary practices
Source: PLoS One. 2020 Sep 1;15(9):e0238364. doi: 10.1371/journal.pone.0238364 (PMC7462311; doi:10.1371/journal.pone.0238364)
Supplement: S1 Table — (DOCX) [file pone.0238364.s002.docx]

**Table S1. Quantile regressions for Bihar, 2015-16**

| **Background variables** |  | | | | |
| --- | --- | --- | --- | --- | --- |
|  | **10th Quintile** | **25th Quintile** | **Median (50th Quintile)** | **75th Quintile** | **90th Quintile** |
| **Size of child at birth (Ref: Average)** |  |  |  |  |  |
| Large | 0.09 (-0.01, 0.19) | 0.06 (0, 1.85) | 0.06 (0, 0.12) | 0.04 (-0.04, 0.12) | 0.11 (0, 0.22) |
| Small | -0.32***(-0.43, -0.21) | -0.22***(-0.3, -5.9) | -0.24***(-0.3, -0.17) | -0.14***(-0.23, -0.06) | -0.09 (-0.22, 0.03) |
| **Age of child (Ref: 0-6 months)** |  |  |  |  |  |
| 6 months-1 year | -0.64***(-0.81, -0.48) | -0.62***(-0.73, -11.04) | -0.7***(-0.79, -0.6) | -0.64***(-0.77, -0.52) | -0.48***(-0.66, -0.3) |
| 1-3 years | -0.96***(-1.08, -0.84) | -1.23***(-1.31, -29.51) | -1.4***(-1.47, -1.32) | -1.45***(-1.54, -1.36) | -1.39***(-1.52, -1.25) |
| 3-5 years | -0.89***(-1.02, -0.77) | -1.15***(-1.23, -27.36) | -1.37***(-1.44, -1.3) | -1.59***(-1.68, -1.5) | -1.82***(-1.95, -1.68) |
| **Sex of child (Ref: Male)** |  |  |  |  |  |
| Female | 0.07*(0, 0.15) | 0.02 (-0.03, 0.96) | -0.03 (-0.07, 0.02) | 0.04 (-0.02, 0.09) | 0.1*(0.02, 0.18) |
| **Birth order (Ref: 1)** |  |  |  |  |  |
| 2 | -0.11*(-0.21, -0.01) | -0.04 (-0.11, -1.05) | -0.06*(-0.12, 0) | -0.04 (-0.12, 0.03) | -0.06 (-0.18, 0.05) |
| 3+ | -0.2***(-0.31, -0.1) | -0.17***(-0.24, -4.79) | -0.13***(-0.19, -0.07) | -0.09*(-0.16, -0.01) | -0.09 (-0.2, 0.03) |
| **Child morbidity (Ref: No disease)** |  |  |  |  |  |
| had at least one disease | -0.02 (-0.13, 0.09) | -0.01 (-0.09, -0.34) | -0.07*(-0.14, -0.01) | -0.12***(-0.2, -0.04) | -0.23***(-0.35, -0.11) |
| **Mother's Body mass index (Ref: Underweight)** |  |  |  |  |  |
| Normal | 0.04 (-0.04, 0.12) | 0.06*(0.01, 2.28) | 0.11***(0.06, 0.16) | 0.14***(0.08, 0.2) | 0.24***(0.15, 0.33) |
| Overweight/obese | 0.31***(0.16, 0.46) | 0.32***(0.22, 6.13) | 0.37***(0.28, 0.46) | 0.38***(0.27, 0.5) | 0.41***(0.24, 0.58) |
| **Education of mother (Ref: No education)** |  |  |  |  |  |
| Primary | 0.26***(0.15, 0.38) | 0.22***(0.14, 5.49) | 0.11***(0.04, 0.18) | 0.07 (-0.01, 0.16) | -0.07 (-0.2, 0.06) |
| Secondary | 0.39***(0.29, 0.49) | 0.36***(0.29, 10.67) | 0.32***(0.27, 0.38) | 0.22***(0.15, 0.3) | 0.07 (-0.04, 0.17) |
| Higher | 0.66***(0.45, 0.87) | 0.59***(0.44, 8.15) | 0.52***(0.4, 0.65) | 0.47***(0.32, 0.63) | 0.23 (0, 0.46) |
| **Mother's age at birth (Ref: Below 20 years)** |  |  |  |  |  |
| 20-29 years | 0.31***(0.16, 0.45) | 0.16***(0.07, 3.28) | 0.13***(0.04, 0.22) | 0.07 (-0.03, 0.18) | -0.01 (-0.17, 0.15) |
| Above 30 years | 0.27***(0.1, 0.45) | 0.14*(0.03, 2.38) | 0.17***(0.07, 0.28) | 0.16*(0.03, 0.29) | 0.19 (0, 0.39) |
| **Child Nutrition Score at PSU** | -0.01 (-0.05, 0.03) | -0.01 (-0.04, -1.06) | -0.01 (-0.03, 0.02) | -0.03*(-0.06, 0) | -0.05*(-0.09, -0.01) |
| **Stool disposal (Ref: Safely disposed)** |  |  |  |  |  |
| Not safely disposed | -0.02 (-0.12, 0.08) | -0.05 (-0.12, -1.52) | -0.09***(-0.16, -0.03) | -0.15***(-0.23, -0.07) | -0.27***(-0.38, -0.15) |
| **Percentage of households that openly defecates in a PSU** | -0.14 (-0.39, 0.1) | -0.16 (-0.33, -1.91) | -0.04 (-0.19, 0.11) | 0.25***(0.06, 0.43) | 0.4***(0.13, 0.67) |
| **Place of residence (Ref: Urban)** |  |  |  |  |  |
| Rural | 0.03 (-0.11, 0.18) | 0 (-0.09, 0.02) | 0 (-0.08, 0.09) | -0.07 (-0.17, 0.04) | -0.11 (-0.27, 0.04) |
| **Religion (Ref: Hindus)** |  |  |  |  |  |
| Non-Hindus | -0.14***(-0.25, -0.04) | -0.14***(-0.21, -3.91) | -0.09***(-0.16, -0.03) | -0.08 (-0.15, 0) | -0.09 (-0.21, 0.02) |
| **Social class (Ref: SC/ST)** |  |  |  |  |  |
| OBC | 0.18***(0.09, 0.27) | 0.17***(0.11, 5.59) | 0.15***(0.1, 0.21) | 0.14***(0.07, 0.21) | 0.13***(0.03, 0.23) |
| Others | 0.33***(0.2, 0.46) | 0.36***(0.27, 7.9) | 0.42***(0.34, 0.5) | 0.46***(0.36, 0.56) | 0.48***(0.34, 0.63) |
| **Wealth Index (Ref: Poor)** |  |  |  |  |  |
| Middle | 0.49***(0.32, 0.65) | 0.47***(0.36, 8.29) | 0.52***(0.42, 0.62) | 0.61***(0.49, 0.74) | 0.61***(0.43, 0.79) |
| Rich | 0.49***(0.32, 0.65) | 0.47***(0.36, 8.29) | 0.52***(0.42, 0.62) | 0.61***(0.49, 0.74) | 0.61***(0.43, 0.79) |
| **Constant** | -3.35***(-3.67, -3.02) | -2.06***(-2.28, -18.35) | -1.05***(-1.25, -0.86) | -0.02 (-0.27, 0.23) | 1.25***(0.89, 1.61) |
