# Supplementary material for: Disparity in childhood stunting in India: Relative importance of community-level nutrition and sanitary practices
Source: PLoS One. 2020 Sep 1;15(9):e0238364. doi: 10.1371/journal.pone.0238364 (PMC7462311; doi:10.1371/journal.pone.0238364)
Supplement: S2 Table — (DOCX) [file pone.0238364.s003.docx]

**Table S2. Quantile regressions for Uttar Pradesh, 2015-16**

| **Background variables** |  | | | | |
| --- | --- | --- | --- | --- | --- |
|  | **10th Quintile** | **25th Quintile** | **Median (50th Quintile)** | **75th Quintile** | **90th Quintile** |
| **Size of child at birth (Ref: Average)** |  |  |  |  |  |
| Large | 0 (-0.08, 0.09) | 0.1***(0.04, 3.33) | 0.11***(0.05, 0.16) | 0.17***(0.11, 0.24) | 0.24***(0.15, 0.34) |
| Small | -0.32***(-0.39, -0.25) | -0.26***(-0.31, -9.82) | -0.29***(-0.34, -0.24) | -0.27***(-0.33, -0.22) | -0.27***(-0.35, -0.18) |
| **Age of child (Ref: 0-6 months)** |  |  |  |  |  |
| 6 months-1 year | -0.25***(-0.36, -0.14) | -0.41***(-0.49, -9.83) | -0.46***(-0.53, -0.38) | -0.51***(-0.59, -0.42) | -0.57***(-0.7, -0.44) |
| 1-3 years | -0.84***(-0.92, -0.76) | -1.16***(-1.22, -38.59) | -1.36***(-1.41, -1.3) | -1.51***(-1.58, -1.45) | -1.58***(-1.68, -1.49) |
| 3-5 years | -0.65***(-0.73, -0.57) | -1***(-1.06, -32.93) | -1.26***(-1.31, -1.2) | -1.5***(-1.56, -1.43) | -1.71***(-1.81, -1.62) |
| **Sex of child (Ref: Male)** |  |  |  |  |  |
| Female | 0.05 (0, 0.1) | 0.04*(0, 2.06) | 0.02 (-0.02, 0.05) | 0 (-0.04, 0.04) | 0.06 (0, 0.12) |
| **Birth order (Ref: 1)** |  |  |  |  |  |
| 2 | -0.1***(-0.16, -0.03) | -0.09***(-0.14, -3.44) | -0.1***(-0.15, -0.06) | -0.05 (-0.1, 0) | -0.04 (-0.12, 0.04) |
| 3+ | -0.27***(-0.34, -0.2) | -0.2***(-0.25, -7.6) | -0.17***(-0.22, -0.13) | -0.14***(-0.2, -0.09) | -0.05 (-0.13, 0.03) |
| **Child morbidity (Ref: No disease)** |  |  |  |  |  |
| had at least one disease | 0.06*(0, 0.13) | 0.03 (-0.02, 1.13) | -0.03 (-0.07, 0.01) | -0.02 (-0.07, 0.02) | -0.08*(-0.15, -0.01) |
| **Mother's Body mass index (Ref: Underweight)** |  |  |  |  |  |
| Normal | 0.11***(0.04, 0.17) | 0.16***(0.12, 7.12) | 0.17***(0.13, 0.21) | 0.18***(0.13, 0.23) | 0.26***(0.18, 0.33) |
| Overweight/obese | 0.36***(0.27, 0.45) | 0.37***(0.3, 11.13) | 0.33***(0.27, 0.39) | 0.27***(0.2, 0.34) | 0.27***(0.17, 0.38) |
| **Education of mother (Ref: No education)** |  |  |  |  |  |
| Primary | 0.17***(0.1, 0.25) | 0.1***(0.04, 3.41) | 0.1***(0.05, 0.16) | 0.02 (-0.04, 0.08) | 0 (-0.09, 0.1) |
| Secondary | 0.34***(0.27, 0.4) | 0.29***(0.24, 11.89) | 0.27***(0.22, 0.31) | 0.19***(0.14, 0.24) | 0.19***(0.11, 0.27) |
| Higher | 0.66***(0.56, 0.76) | 0.61***(0.53, 16.02) | 0.6***(0.53, 0.67) | 0.56***(0.48, 0.64) | 0.62***(0.5, 0.74) |
| **Mother's age at birth (Ref: Below 20 years)** |  |  |  |  |  |
| 20-29 years | 0.15*(0.01, 0.28) | 0.09 (-0.01, 1.74) | 0.1*(0.01, 0.19) | 0.13*(0.02, 0.23) | 0.2*(0.04, 0.36) |
| Above 30 years | 0.23***(0.08, 0.38) | 0.13*(0.02, 2.25) | 0.17***(0.07, 0.28) | 0.24***(0.12, 0.35) | 0.37***(0.19, 0.54) |
| **Child Nutrition Score at PSU** | 0.04***(0.01, 0.06) | 0.02*(0, 2.22) | 0.01 (-0.01, 0.03) | 0 (-0.02, 0.02) | -0.01 (-0.04, 0.02) |
| **Stool disposal (Ref: Safely disposed)** |  |  |  |  |  |
| Not safely disposed | -0.13***(-0.19, -0.07) | -0.1***(-0.15, -4.49) | -0.1***(-0.14, -0.05) | -0.06***(-0.11, -0.02) | -0.06 (-0.14, 0.01) |
| **Percentage of households that openly defecates in a PSU** | -0.62***(-0.75, -0.48) | -0.54***(-0.64, -10.62) | -0.39***(-0.48, -0.3) | -0.28***(-0.39, -0.17) | -0.29***(-0.45, -0.13) |
| **Place of residence (Ref: Urban)** |  |  |  |  |  |
| Rural | 0.24***(0.17, 0.32) | 0.21***(0.15, 7.09) | 0.14***(0.09, 0.2) | 0.08***(0.02, 0.14) | 0.02 (-0.07, 0.11) |
| **Religion (Ref: Hindus)** |  |  |  |  |  |
| Non-Hindus | -0.13***(-0.2, -0.06) | -0.13***(-0.18, -5.12) | -0.13***(-0.18, -0.09) | -0.12***(-0.17, -0.07) | -0.17***(-0.25, -0.09) |
| **Social class (Ref: SC/ST)** |  |  |  |  |  |
| OBC | 0.12***(0.06, 0.18) | 0.1***(0.06, 4.39) | 0.1***(0.06, 0.14) | 0.09***(0.04, 0.14) | 0.1***(0.03, 0.18) |
| Others | 0.16***(0.08, 0.24) | 0.23***(0.17, 7.52) | 0.24***(0.19, 0.3) | 0.26***(0.2, 0.33) | 0.33***(0.23, 0.42) |
| **Wealth Index (Ref: Poor)** |  |  |  |  |  |
| Middle | 0.43***(0.35, 0.51) | 0.38***(0.32, 12.38) | 0.33***(0.28, 0.39) | 0.31***(0.24, 0.37) | 0.14***(0.04, 0.24) |
| Rich | 0.43***(0.35, 0.51) | 0.38***(0.32, 12.38) | 0.33***(0.28, 0.39) | 0.31***(0.24, 0.37) | 0.14***(0.04, 0.24) |
| **Constant** | -3.29***(-3.52, -3.07) | -2.13***(-2.29, -25.77) | -1.07***(-1.22, -0.92) | -0.05 (-0.22, 0.13) | 0.94***(0.68, 1.2) |
