# Supplementary material for: Disparity in childhood stunting in India: Relative importance of community-level nutrition and sanitary practices
Source: PLoS One. 2020 Sep 1;15(9):e0238364. doi: 10.1371/journal.pone.0238364 (PMC7462311; doi:10.1371/journal.pone.0238364)
Supplement: S3 Table — (DOCX) [file pone.0238364.s004.docx]

**Table S3. Quantile regressions for Madhya Pradesh, 2015-16**

| **Background variables** |  | | | | |
| --- | --- | --- | --- | --- | --- |
|  | **10th Quintile** | **25th Quintile** | **Median (50th Quintile)** | **75th Quintile** | **90th Quintile** |
| **Size of child at birth (Ref: Average)** |  |  |  |  |  |
| Large | -0.08 (-0.17, 0.01) | -0.05 (-0.12, -1.62) | -0.04 (-0.1, 0.02) | 0 (-0.07, 0.07) | -0.01 (-0.13, 0.11) |
| Small | -0.3***(-0.41, -0.19) | -0.31***(-0.39, -7.86) | -0.26***(-0.33, -0.19) | -0.22***(-0.3, -0.13) | -0.17*(-0.32, -0.03) |
| **Age of child (Ref: 0-6 months)** |  |  |  |  |  |
| 6 months-1 year | -0.41***(-0.57, -0.25) | -0.56***(-0.67, -9.94) | -0.52***(-0.63, -0.42) | -0.57***(-0.7, -0.45) | -0.53***(-0.74, -0.32) |
| 1-3 years | -0.81***(-0.93, -0.69) | -1.21***(-1.29, -28.81) | -1.36***(-1.43, -1.28) | -1.45***(-1.54, -1.36) | -1.48***(-1.64, -1.33) |
| 3-5 years | -0.6***(-0.72, -0.48) | -1.05***(-1.13, -24.78) | -1.29***(-1.37, -1.22) | -1.54***(-1.63, -1.44) | -1.83***(-1.98, -1.67) |
| **Sex of child (Ref: Male)** |  |  |  |  |  |
| Female | 0.12***(0.04, 0.19) | 0.09***(0.04, 3.62) | 0.03 (-0.02, 0.08) | 0.04 (-0.02, 0.09) | 0.11*(0.01, 0.2) |
| **Birth order (Ref: 1)** |  |  |  |  |  |
| 2 | -0.15***(-0.24, -0.06) | -0.11***(-0.17, -3.48) | -0.02 (-0.08, 0.04) | 0 (-0.07, 0.07) | 0 (-0.12, 0.11) |
| 3+ | -0.16***(-0.27, -0.06) | -0.17***(-0.23, -4.64) | -0.1***(-0.17, -0.04) | -0.12***(-0.2, -0.04) | -0.09 (-0.22, 0.04) |
| **Child morbidity (Ref: No disease)** |  |  |  |  |  |
| had at least one disease | 0.08 (-0.03, 0.18) | 0.01 (-0.06, 0.34) | 0.01 (-0.06, 0.07) | -0.02 (-0.1, 0.06) | -0.09 (-0.23, 0.04) |
| **Mother's Body mass index (Ref: Underweight)** |  |  |  |  |  |
| Normal | 0.05 (-0.03, 0.13) | 0.09***(0.04, 3.31) | 0.13***(0.08, 0.18) | 0.19***(0.13, 0.25) | 0.31***(0.21, 0.41) |
| Overweight/obese | 0.36***(0.21, 0.51) | 0.29***(0.19, 5.62) | 0.28***(0.19, 0.38) | 0.33***(0.22, 0.45) | 0.43***(0.24, 0.63) |
| **Education of mother (Ref: No education)** |  |  |  |  |  |
| Primary | 0.26***(0.15, 0.36) | 0.19***(0.12, 5.21) | 0.09***(0.02, 0.16) | -0.01 (-0.1, 0.07) | -0.14*(-0.27, 0) |
| Secondary | 0.41***(0.31, 0.51) | 0.3***(0.24, 8.99) | 0.19***(0.13, 0.26) | 0.05 (-0.02, 0.13) | -0.11 (-0.24, 0.01) |
| Higher | 0.66***(0.47, 0.84) | 0.59***(0.46, 9.13) | 0.5***(0.38, 0.62) | 0.33***(0.19, 0.47) | 0.13 (-0.11, 0.36) |
| **Mother's age at birth (Ref: Below 20 years)** |  |  |  |  |  |
| 20-29 years | 0.24***(0.09, 0.39) | 0.2***(0.1, 3.86) | 0.12*(0.02, 0.21) | 0.04 (-0.07, 0.16) | 0.13 (-0.06, 0.33) |
| Above 30 years | 0.28***(0.09, 0.47) | 0.23***(0.11, 3.58) | 0.22***(0.1, 0.34) | 0.26***(0.12, 0.41) | 0.43***(0.18, 0.67) |
| **Child Nutrition Score at PSU** | 0.03 (-0.01, 0.06) | 0.02*(0, 2.05) | 0.01 (-0.02, 0.03) | 0 (-0.02, 0.03) | -0.04 (-0.08, 0) |
| **Stool disposal (Ref: Safely disposed)** |  |  |  |  |  |
| Not safely disposed | -0.06 (-0.15, 0.03) | -0.07*(-0.13, -2.1) | -0.05 (-0.11, 0.01) | -0.08*(-0.15, -0.01) | 0 (-0.11, 0.12) |
| **Percentage of households that openly defecates in a PSU** | -0.23*(-0.43, -0.04) | -0.27***(-0.41, -3.97) | -0.19***(-0.31, -0.06) | -0.35***(-0.5, -0.19) | -0.36***(-0.62, -0.11) |
| **Place of residence (Ref: Urban)** |  |  |  |  |  |
| Rural | 0.16***(0.04, 0.28) | 0.2***(0.12, 4.84) | 0.2***(0.12, 0.28) | 0.25***(0.15, 0.34) | 0.26***(0.11, 0.41) |
| **Religion (Ref: Hindus)** |  |  |  |  |  |
| Non-Hindus | -0.11 (-0.25, 0.03) | -0.05 (-0.15, -1.07) | -0.08 (-0.17, 0.01) | -0.06 (-0.17, 0.05) | -0.14 (-0.32, 0.04) |
| **Social class (Ref: SC/ST)** |  |  |  |  |  |
| OBC | 0.17***(0.09, 0.25) | 0.11***(0.05, 3.67) | 0.14***(0.09, 0.2) | 0.05 (-0.01, 0.12) | -0.03 (-0.14, 0.08) |
| Others | 0.27***(0.14, 0.4) | 0.23***(0.14, 5.16) | 0.24***(0.16, 0.32) | 0.13***(0.03, 0.23) | 0.03 (-0.14, 0.19) |
| **Wealth Index (Ref: Poor)** |  |  |  |  |  |
| Middle | 0.25***(0.12, 0.38) | 0.29***(0.2, 6.49) | 0.3***(0.22, 0.38) | 0.24***(0.15, 0.34) | 0.32***(0.16, 0.48) |
| Rich | 0.25***(0.12, 0.38) | 0.29***(0.2, 6.49) | 0.3***(0.22, 0.38) | 0.24***(0.15, 0.34) | 0.32***(0.16, 0.48) |
| **Constant** | -3.6***(-3.9, -3.3) | -2.23***(-2.43, -21.07) | -1.09***(-1.28, -0.9) | 0.28*(0.05, 0.52) | 1.48***(1.09, 1.87) |
