# Supplementary material for: Disparity in childhood stunting in India: Relative importance of community-level nutrition and sanitary practices
Source: PLoS One. 2020 Sep 1;15(9):e0238364. doi: 10.1371/journal.pone.0238364 (PMC7462311; doi:10.1371/journal.pone.0238364)
Supplement: S4 Table — (DOCX) [file pone.0238364.s005.docx]

**Table S4. Quantile regressions for Gujarat, 2015-16**

| **Background variables** |  | | | | |
| --- | --- | --- | --- | --- | --- |
|  | **10th Quintile** | **25th Quintile** | **Median (50th Quintile)** | **75th Quintile** | **90th Quintile** |
| **Size of child at birth (Ref: Average)** |  |  |  |  |  |
| Large | 0.10 (-0.06, 0.25) | 0.09 (-0.03, 1.52) | 0.12*(0.02, 0.22) | 0.14*(0, 0.28) | 0.12 (-0.09, 0.32) |
| Small | -0.21*(-0.39, -0.02) | -0.24***(-0.38, -3.19) | -0.23***(-0.35, -0.1) | -0.22***(-0.39, -0.06) | -0.21 (-0.46, 0.04) |
| **Age of child (Ref: 0-6 months)** |  |  |  |  |  |
| 6 months-1 year | -0.49***(-0.78, -0.2) | -0.58***(-0.81, -5.06) | -0.63***(-0.82, -0.44) | -0.46***(-0.72, -0.2) | -0.47*(-0.85, -0.08) |
| 1-3 years | -1.09***(-1.31, -0.88) | -1.42***(-1.58, -16.72) | -1.48***(-1.62, -1.33) | -1.46***(-1.65, -1.27) | -1.59***(-1.87, -1.3) |
| 3-5 years | -0.83***(-1.05, -0.62) | -1.2***(-1.37, -14.16) | -1.43***(-1.57, -1.29) | -1.64***(-1.84, -1.45) | -2.16***(-2.45, -1.88) |
| **Sex of child (Ref: Male)** |  |  |  |  |  |
| Female | 0.17***(0.04, 0.3) | 0.11*(0.01, 2.18) | 0.11*(0.02, 0.19) | 0.06 (-0.05, 0.18) | 0.06 (-0.11, 0.24) |
| **Birth order (Ref: 1)** |  |  |  |  |  |
| 2 | -0.14 (-0.3, 0.02) | -0.09 (-0.21, -1.5) | -0.08 (-0.19, 0.02) | -0.15*(-0.29, -0.01) | -0.13 (-0.33, 0.08) |
| 3+ | -0.34***(-0.53, -0.15) | -0.21***(-0.35, -2.83) | -0.16***(-0.29, -0.04) | -0.24***(-0.41, -0.07) | -0.09 (-0.34, 0.16) |
| **Child morbidity (Ref: No disease)** |  |  |  |  |  |
| had at least one disease | 0.17 (-0.04, 0.37) | 0.05 (-0.11, 0.65) | 0.02 (-0.12, 0.15) | -0.09 (-0.27, 0.1) | -0.11 (-0.39, 0.16) |
| **Mother's Body mass index (Ref: Underweight)** |  |  |  |  |  |
| Normal | 0.1 (-0.05, 0.25) | 0.1 (-0.01, 1.76) | 0.15***(0.05, 0.25) | 0.26***(0.12, 0.39) | 0.51***(0.31, 0.71) |
| Overweight/obese | 0.26*(0.05, 0.48) | 0.29***(0.13, 3.44) | 0.38***(0.24, 0.52) | 0.4***(0.21, 0.59) | 0.48***(0.2, 0.77) |
| **Education of mother (Ref: No education)** |  |  |  |  |  |
| Primary | 0.06 (-0.15, 0.27) | 0.11 (-0.06, 1.29) | 0.03 (-0.11, 0.17) | 0.09 (-0.1, 0.28) | -0.02 (-0.3, 0.27) |
| Secondary | 0.18*(0, 0.36) | 0.13 (-0.01, 1.8) | 0.07 (-0.04, 0.19) | -0.01 (-0.17, 0.15) | -0.21 (-0.45, 0.02) |
| Higher | 0.5***(0.19, 0.81) | 0.43***(0.19, 3.52) | 0.3***(0.09, 0.5) | 0.15 (-0.13, 0.42) | 0.31 (-0.1, 0.71) |
| **Mother's age at birth (Ref: Below 20 years)** |  |  |  |  |  |
| 20-29 years | 0.16 (-0.1, 0.42) | 0.16 (-0.05, 1.51) | 0.19*(0.02, 0.36) | 0.31***(0.07, 0.54) | 0.37*(0.02, 0.72) |
| Above 30 years | 0.16 (-0.16, 0.47) | 0.12 (-0.12, 0.98) | 0.19 (-0.02, 0.4) | 0.39***(0.11, 0.68) | 0.32 (-0.1, 0.74) |
| **Child Nutrition Score at PSU** | -0.02 (-0.08, 0.04) | -0.02 (-0.07, -1.01) | 0.01 (-0.03, 0.05) | 0.03 (-0.03, 0.08) | 0.02 (-0.06, 0.1) |
| **Stool disposal (Ref: Safely disposed)** |  |  |  |  |  |
| Not safely disposed | -0.01 (-0.17, 0.15) | 0 (-0.13, -0.03) | 0.05 (-0.06, 0.15) | 0.04 (-0.11, 0.18) | 0.17 (-0.04, 0.38) |
| **Percentage of households that openly defecates in a PSU** | -0.02 (-0.32, 0.29) | 0.07 (-0.16, 0.61) | -0.06 (-0.26, 0.14) | -0.18 (-0.45, 0.09) | -0.02 (-0.42, 0.38) |
| **Place of residence (Ref: Urban)** |  |  |  |  |  |
| Rural | -0.02 (-0.2, 0.17) | 0 (-0.14, 0.04) | 0.07 (-0.05, 0.19) | 0.08 (-0.08, 0.24) | -0.01 (-0.25, 0.23) |
| **Religion (Ref: Hindus)** |  |  |  |  |  |
| Non-Hindus | 0.07 (-0.15, 0.28) | 0.02 (-0.15, 0.25) | 0.01 (-0.13, 0.15) | -0.02 (-0.21, 0.17) | 0.26 (-0.02, 0.55) |
| **Social class (Ref: SC/ST)** |  |  |  |  |  |
| OBC | 0.02 (-0.14, 0.18) | 0 (-0.12, -0.01) | 0.06 (-0.04, 0.16) | 0.07 (-0.07, 0.21) | 0.11 (-0.1, 0.31) |
| Others | 0.1 (-0.1, 0.31) | 0.02 (-0.14, 0.26) | 0.1 (-0.03, 0.23) | 0.12 (-0.06, 0.31) | 0.1 (-0.17, 0.37) |
| **Wealth Index (Ref: Poor)** |  |  |  |  |  |
| Middle | 0.48***(0.26, 0.7) | 0.59***(0.42, 6.74) | 0.56***(0.41, 0.7) | 0.4***(0.2, 0.6) | 0.57***(0.27, 0.87) |
| Rich | 0.48***(0.26, 0.7) | 0.59***(0.42, 6.74) | 0.56***(0.41, 0.7) | 0.4***(0.2, 0.6) | 0.57***(0.27, 0.87) |
| **Constant** | -3.08***(-3.58, -2.58) | -1.93***(-2.32, -9.76) | -1.08***(-1.41, -0.75) | -0.1 (-0.54, 0.35) | 0.99***(0.33, 1.66) |
