# Supplementary material for: Disparity in childhood stunting in India: Relative importance of community-level nutrition and sanitary practices
Source: PLoS One. 2020 Sep 1;15(9):e0238364. doi: 10.1371/journal.pone.0238364 (PMC7462311; doi:10.1371/journal.pone.0238364)
Supplement: S5 Table — (DOCX) [file pone.0238364.s006.docx]

**Table S5. Quantile regressions for Punjab, 2015-16**

| **Background variables** |  | | | | |
| --- | --- | --- | --- | --- | --- |
|  | **10th Quintile** | **25th Quintile** | **Median (50th Quintile)** | **75th Quintile** | **90th Quintile** |
| **Size of child at birth (Ref: Average)** |  |  |  |  |  |
| Large | -0.01 (-0.22, 0.2) | 0.06 (-0.08, 0.89) | 0.08 (-0.05, 0.22) | 0.13 (-0.02, 0.27) | 0.19 (-0.08, 0.46) |
| Small | -0.27*(-0.51, -0.03) | -0.3***(-0.46, -3.78) | -0.26***(-0.42, -0.11) | -0.28***(-0.44, -0.11) | -0.09 (-0.39, 0.22) |
| **Age of child (Ref: 0-6 months)** |  |  |  |  |  |
| 6 months-1 year | -0.05 (-0.39, 0.29) | -0.11 (-0.33, -0.94) | -0.08 (-0.3, 0.13) | -0.17 (-0.4, 0.07) | -0.27 (-0.7, 0.16) |
| 1-3 years | -0.53***(-0.77, -0.28) | -0.62***(-0.78, -7.43) | -0.82***(-0.98, -0.66) | -0.86***(-1.04, -0.69) | -0.93***(-1.25, -0.62) |
| 3-5 years | -0.29*(-0.54, -0.04) | -0.47***(-0.64, -5.6) | -0.73***(-0.89, -0.57) | -0.83***(-1, -0.66) | -1.11***(-1.43, -0.79) |
| **Sex of child (Ref: Male)** |  |  |  |  |  |
| Female | 0.04 (-0.11, 0.18) | 0.03 (-0.06, 0.65) | 0.03 (-0.07, 0.12) | 0.08 (-0.02, 0.18) | 0.03 (-0.16, 0.22) |
| **Birth order (Ref: 1)** |  |  |  |  |  |
| 2 | -0.13 (-0.3, 0.04) | -0.16***(-0.27, -2.81) | -0.15***(-0.26, -0.04) | -0.08 (-0.2, 0.04) | -0.09 (-0.3, 0.13) |
| 3+ | -0.28*(-0.51, -0.05) | -0.24***(-0.39, -3.06) | -0.24***(-0.38, -0.09) | -0.19*(-0.35, -0.03) | -0.16 (-0.45, 0.13) |
| **Child morbidity (Ref: No disease)** |  |  |  |  |  |
| had at least one disease | -0.01 (-0.21, 0.18) | 0.08 (-0.05, 1.22) | 0.05 (-0.07, 0.18) | -0.02 (-0.16, 0.11) | 0.1 (-0.15, 0.35) |
| **Mother's Body mass index (Ref: Underweight)** |  |  |  |  |  |
| Normal | 0.19 (-0.04, 0.42) | 0.25***(0.1, 3.26) | 0.19***(0.05, 0.34) | 0.25***(0.1, 0.41) | 0.32*(0.03, 0.61) |
| Overweight/obese | 0.44***(0.19, 0.7) | 0.42***(0.25, 4.89) | 0.31***(0.15, 0.47) | 0.37***(0.19, 0.55) | 0.36*(0.03, 0.69) |
| **Education of mother (Ref: No education)** |  |  |  |  |  |
| Primary | 0.11 (-0.18, 0.4) | 0.12 (-0.07, 1.26) | 0.17 (-0.01, 0.36) | 0.2 (0, 0.4) | 0.32 (-0.05, 0.69) |
| Secondary | 0.08 (-0.16, 0.33) | 0.13 (-0.03, 1.61) | 0.19*(0.04, 0.35) | 0.32***(0.15, 0.49) | 0.28 (-0.03, 0.59) |
| Higher | 0.24 (-0.07, 0.56) | 0.26*(0.05, 2.46) | 0.4***(0.2, 0.6) | 0.51***(0.29, 0.73) | 0.53***(0.12, 0.93) |
| **Mother's age at birth (Ref: Below 20 years)** |  |  |  |  |  |
| 20-29 years | 0.1 (-0.34, 0.54) | 0.35*(0.06, 2.36) | 0.32*(0.04, 0.6) | 0.21 (-0.09, 0.52) | 0.48 (-0.08, 1.04) |
| Above 30 years | 0.13 (-0.35, 0.61) | 0.42***(0.1, 2.59) | 0.4***(0.1, 0.71) | 0.24 (-0.09, 0.58) | 0.51 (-0.1, 1.12) |
| **Child Nutrition Score at PSU** | 0.03 (-0.03, 0.09) | 0.01 (-0.03, 0.61) | 0 (-0.04, 0.04) | 0.01 (-0.04, 0.05) | 0.01 (-0.07, 0.09) |
| **Stool disposal (Ref: Safely disposed)** |  |  |  |  |  |
| Not safely disposed | 0.06 (-0.15, 0.26) | 0.02 (-0.12, 0.23) | 0.03 (-0.1, 0.17) | 0.03 (-0.11, 0.17) | -0.06 (-0.32, 0.2) |
| **Percentage of households that openly defecates in a PSU** | -0.46 (-0.93, 0.01) | -0.2 (-0.51, -1.26) | -0.3 (-0.6, 0) | -0.14 (-0.46, 0.19) | -0.42 (-1.02, 0.18) |
| **Place of residence (Ref: Urban)** |  |  |  |  |  |
| Rural | 0.27***(0.1, 0.44) | 0.18***(0.07, 3.16) | 0.16***(0.05, 0.27) | -0.02 (-0.14, 0.1) | -0.15 (-0.36, 0.07) |
| **Religion (Ref: Hindus)** |  |  |  |  |  |
| Non-Hindus | 0.15 (-0.02, 0.31) | 0.17***(0.06, 3.11) | 0.13*(0.02, 0.24) | 0.21***(0.09, 0.32) | 0.29***(0.08, 0.5) |
| **Social class (Ref: SC/ST)** |  |  |  |  |  |
| OBC | 0.18 (-0.03, 0.38) | 0.17*(0.03, 2.44) | 0.2***(0.07, 0.33) | 0.17*(0.03, 0.32) | 0.06 (-0.2, 0.32) |
| Others | 0.12 (-0.06, 0.31) | 0.23***(0.11, 3.75) | 0.27***(0.15, 0.39) | 0.24***(0.11, 0.37) | 0.13 (-0.11, 0.36) |
| **Wealth Index (Ref: Poor)** |  |  |  |  |  |
| Middle | 0.57***(0.2, 0.94) | 0.45***(0.21, 3.64) | 0.26*(0.02, 0.49) | 0.07 (-0.19, 0.33) | 0.16 (-0.31, 0.63) |
| Rich | 0.57***(0.2, 0.94) | 0.45***(0.21, 3.64) | 0.26*(0.02, 0.49) | 0.07 (-0.19, 0.33) | 0.16 (-0.31, 0.63) |
| **Constant** | -3.64***(-4.36, -2.93) | -2.96***(-3.43, -12.45) | -1.63***(-2.08, -1.17) | -0.61*(-1.1, -0.12) | 0.15 (-0.75, 1.05) |
