# Supplementary material for: Disparity in childhood stunting in India: Relative importance of community-level nutrition and sanitary practices
Source: PLoS One. 2020 Sep 1;15(9):e0238364. doi: 10.1371/journal.pone.0238364 (PMC7462311; doi:10.1371/journal.pone.0238364)
Supplement: S6 Table — (DOCX) [file pone.0238364.s007.docx]

**Table S6. Quantile regressions for Odisha, 2015-16**

| **Background variables** |  | | | | |
| --- | --- | --- | --- | --- | --- |
|  | **10th Quintile** | **25th Quintile** | **Median (50th Quintile)** | **75th Quintile** | **90th Quintile** |
| **Size of child at birth (Ref: Average)** |  |  |  |  |  |
| Large | 0 (-0.11, 0.12) | 0.07 (-0.01, 1.72) | 0.1***(0.03, 0.18) | 0.09*(0, 0.18) | 0.05 (-0.1, 0.2) |
| Small | -0.2***(-0.34, -0.05) | -0.19***(-0.3, -3.65) | -0.18***(-0.27, -0.08) | -0.24***(-0.35, -0.13) | -0.21*(-0.4, -0.02) |
| **Age of child (Ref: 0-6 months)** |  |  |  |  |  |
| 6 months-1 year | 0.08 (-0.14, 0.3) | -0.04 (-0.2, -0.53) | -0.14*(-0.29, 0) | -0.29***(-0.45, -0.12) | -0.25 (-0.54, 0.04) |
| 1-3 years | -0.26***(-0.42, -0.1) | -0.66***(-0.77, -11.02) | -0.95***(-1.05, -0.84) | -1.17***(-1.29, -1.04) | -1.32***(-1.53, -1.11) |
| 3-5 years | 0.03 (-0.14, 0.19) | -0.48***(-0.59, -7.94) | -0.9***(-1.01, -0.8) | -1.26***(-1.39, -1.14) | -1.67***(-1.88, -1.45) |
| **Sex of child (Ref: Male)** |  |  |  |  |  |
| Female | 0.18***(0.09, 0.28) | 0.04 (-0.03, 1.25) | 0.01 (-0.06, 0.07) | 0.03 (-0.04, 0.1) | 0 (-0.12, 0.13) |
| **Birth order (Ref: 1)** |  |  |  |  |  |
| 2 | -0.02 (-0.13, 0.1) | -0.04 (-0.12, -0.96) | -0.12***(-0.2, -0.05) | -0.1*(-0.19, -0.01) | -0.07 (-0.23, 0.08) |
| 3+ | -0.16*(-0.3, -0.01) | -0.15***(-0.25, -2.82) | -0.25***(-0.35, -0.16) | -0.18***(-0.29, -0.07) | -0.17 (-0.36, 0.02) |
| **Child morbidity (Ref: No disease)** |  |  |  |  |  |
| had at least one disease | -0.04 (-0.18, 0.1) | -0.06 (-0.16, -1.23) | -0.06 (-0.15, 0.03) | 0.03 (-0.07, 0.13) | 0.01 (-0.17, 0.18) |
| **Mother's Body mass index (Ref: Underweight)** |  |  |  |  |  |
| Normal | 0.22***(0.11, 0.33) | 0.17***(0.09, 4.17) | 0.2***(0.13, 0.27) | 0.27***(0.18, 0.35) | 0.32***(0.18, 0.47) |
| Overweight/obese | 0.57***(0.4, 0.75) | 0.43***(0.3, 6.55) | 0.45***(0.33, 0.56) | 0.48***(0.35, 0.61) | 0.54***(0.31, 0.77) |
| **Education of mother (Ref: No education)** |  |  |  |  |  |
| Primary | 0.25***(0.09, 0.41) | 0.27***(0.16, 4.69) | 0.12*(0.02, 0.23) | 0.04 (-0.08, 0.16) | -0.16 (-0.37, 0.05) |
| Secondary | 0.35***(0.22, 0.48) | 0.31***(0.22, 6.39) | 0.21***(0.12, 0.3) | 0.21***(0.11, 0.31) | 0.11 (-0.07, 0.28) |
| Higher | 0.25 (-0.02, 0.52) | 0.45***(0.26, 4.64) | 0.48***(0.31, 0.66) | 0.59***(0.39, 0.79) | 0.34 (-0.01, 0.69) |
| **Mother's age at birth (Ref: Below 20 years)** |  |  |  |  |  |
| 20-29 years | 0.12 (-0.08, 0.32) | 0.09 (-0.06, 1.17) | 0.17***(0.04, 0.3) | 0.12 (-0.03, 0.27) | 0.26*(0, 0.52) |
| Above 30 years | 0.21 (-0.02, 0.44) | 0.17 (0, 1.94) | 0.23***(0.08, 0.38) | 0.23*(0.05, 0.4) | 0.37*(0.07, 0.68) |
| **Child Nutrition Score at PSU** | 0.03 (-0.01, 0.07) | -0.01 (-0.04, -0.99) | -0.02 (-0.05, 0) | -0.01 (-0.04, 0.02) | -0.01 (-0.07, 0.04) |
| **Stool disposal (Ref: Safely disposed)** |  |  |  |  |  |
| Not safely disposed | 0.02 (-0.13, 0.16) | -0.03 (-0.13, -0.52) | -0.07 (-0.16, 0.03) | -0.06 (-0.17, 0.06) | 0.02 (-0.18, 0.21) |
| **Percentage of households that openly defecates in a PSU** | -0.36***(-0.6, -0.11) | -0.13 (-0.3, -1.42) | -0.06 (-0.22, 0.1) | -0.02 (-0.21, 0.16) | -0.04 (-0.36, 0.28) |
| **Place of residence (Ref: Urban)** |  |  |  |  |  |
| Rural | 0.15 (0, 0.31) | 0.18***(0.07, 3.12) | 0.13*(0.03, 0.23) | 0.09 (-0.02, 0.21) | 0.1 (-0.11, 0.3) |
| **Religion (Ref: Hindus)** |  |  |  |  |  |
| Non-Hindus | 0.04 (-0.15, 0.24) | 0.01 (-0.14, 0.08) | 0.04 (-0.09, 0.17) | 0.05 (-0.1, 0.2) | 0.21 (-0.05, 0.47) |
| **Social class (Ref: SC/ST)** |  |  |  |  |  |
| OBC | 0.11 (0, 0.23) | 0.09*(0.01, 2.09) | 0.1***(0.03, 0.18) | 0.07 (-0.01, 0.16) | 0.04 (-0.11, 0.19) |
| Others | 0.35***(0.19, 0.5) | 0.37***(0.26, 6.43) | 0.4***(0.3, 0.51) | 0.32***(0.2, 0.44) | 0.45***(0.25, 0.66) |
| **Wealth Index (Ref: Poor)** |  |  |  |  |  |
| Middle | 0.34***(0.16, 0.51) | 0.45***(0.32, 6.85) | 0.44***(0.32, 0.55) | 0.41***(0.28, 0.55) | 0.35***(0.12, 0.59) |
| Rich | 0.34***(0.16, 0.51) | 0.45***(0.32, 6.85) | 0.44***(0.32, 0.55) | 0.41***(0.28, 0.55) | 0.35***(0.12, 0.59) |
| **Constant** | -3.8***(-4.2, -3.41) | -2.45***(-2.73, -16.99) | -1.19***(-1.45, -0.93) | -0.15 (-0.45, 0.15) | 0.88***(0.37, 1.4) |
