# Supplementary material for: Disparity in childhood stunting in India: Relative importance of community-level nutrition and sanitary practices
Source: PLoS One. 2020 Sep 1;15(9):e0238364. doi: 10.1371/journal.pone.0238364 (PMC7462311; doi:10.1371/journal.pone.0238364)
Supplement: S7 Table — (DOCX) [file pone.0238364.s008.docx]

**Table S7. Quantile regressions for Tamil Nadu, 2015-16**

| **Background variables** |  | | | | |
| --- | --- | --- | --- | --- | --- |
|  | **10th Quintile** | **25th Quintile** | **Median (50th Quintile)** | **75th Quintile** | **90th Quintile** |
| **Size of child at birth (Ref: Average)** |  |  |  |  |  |
| Large | 0.13 (-0.03, 0.3) | 0.07 (-0.04, 1.26) | 0.03 (-0.07, 0.13) | 0.03 (-0.1, 0.15) | 0.08 (-0.14, 0.3) |
| Small | -0.08 (-0.32, 0.17) | -0.20*(-0.37, -2.38) | -0.37***(-0.51, -0.23) | -0.53***(-0.72, -0.35) | -0.52***(-0.84, -0.2) |
| **Age of child (Ref: 0-6 months)** |  |  |  |  |  |
| 6 months-1 year | -0.03 (-0.37, 0.31) | -0.01 (-0.24, -0.11) | -0.40***(-0.59, -0.2) | -0.47***(-0.73, -0.22) | -0.15 (-0.59, 0.29) |
| 1-3 years | -0.1 (-0.35, 0.15) | -0.42***(-0.59, -4.76) | -0.95***(-1.1, -0.81) | -1.27***(-1.46, -1.08) | -0.99***(-1.32, -0.66) |
| 3-5 years | 0.3*(0.04, 0.55) | -0.22*(-0.39, -2.46) | -0.90***(-1.05, -0.75) | -1.38***(-1.57, -1.19) | -1.55***(-1.88, -1.22) |
| **Sex of child (Ref: Male)** |  |  |  |  |  |
| Female | 0.14*(0, 0.29) | 0.09 (-0.01, 1.85) | 0.07 (-0.02, 0.15) | 0.03 (-0.08, 0.14) | -0.03 (-0.22, 0.15) |
| **Birth order (Ref: 1)** |  |  |  |  |  |
| 2 | -0.01 (-0.16, 0.15) | -0.14*(-0.25, -2.54) | -0.1*(-0.2, -0.01) | -0.19***(-0.31, -0.07) | -0.16 (-0.37, 0.04) |
| 3+ | -0.08 (-0.33, 0.16) | -0.17*(-0.34, -2.02) | -0.13 (-0.27, 0.02) | -0.01 (-0.19, 0.18) | -0.03 (-0.35, 0.29) |
| **Child morbidity (Ref: No disease)** |  |  |  |  |  |
| had at least one disease | -0.01 (-0.21, 0.18) | -0.05 (-0.19, -0.76) | -0.1 (-0.21, 0.02) | -0.18*(-0.33, -0.03) | -0.22 (-0.47, 0.04) |
| **Mother's Body mass index (Ref: Underweight)** |  |  |  |  |  |
| Normal | 0.08 (-0.13, 0.29) | 0.07 (-0.07, 0.95) | 0.11 (-0.01, 0.23) | 0.28***(0.12, 0.44) | 0.44***(0.16, 0.71) |
| Overweight/obese | 0.26*(0.03, 0.5) | 0.15 (-0.01, 1.89) | 0.1 (-0.04, 0.23) | 0.14 (-0.04, 0.31) | 0.05 (-0.25, 0.35) |
| **Education of mother (Ref: No education)** |  |  |  |  |  |
| Primary | 0.02 (-0.38, 0.43) | 0.14 (-0.14, 1) | -0.05 (-0.29, 0.19) | -0.13 (-0.44, 0.17) | -0.66*(-1.19, -0.13) |
| Secondary | 0.26 (-0.06, 0.59) | 0.35***(0.13, 3.06) | 0.2*(0.01, 0.4) | 0.12 (-0.13, 0.36) | -0.31 (-0.74, 0.11) |
| Higher | 0.4*(0.03, 0.76) | 0.41***(0.16, 3.19) | 0.33***(0.11, 0.54) | 0.19 (-0.09, 0.46) | -0.17 (-0.65, 0.3) |
| **Mother's age at birth (Ref: Below 20 years)** |  |  |  |  |  |
| 20-29 years | 0.02 (-0.29, 0.32) | -0.1 (-0.31, -0.93) | -0.02 (-0.2, 0.16) | 0.11 (-0.12, 0.34) | 0.24 (-0.17, 0.64) |
| Above 30 years | 0.17 (-0.19, 0.54) | 0.03 (-0.22, 0.24) | 0.04 (-0.18, 0.26) | 0.29*(0.01, 0.56) | 0.28 (-0.2, 0.77) |
| **Child Nutrition Score at PSU** | -0.03 (-0.08, 0.02) | -0.02 (-0.05, -1.28) | -0.01 (-0.04, 0.02) | -0.01 (-0.05, 0.03) | -0.01 (-0.08, 0.05) |
| **Stool disposal (Ref: Safely disposed)** |  |  |  |  |  |
| Not safely disposed | -0.17*(-0.33, 0) | -0.12*(-0.23, -2.1) | -0.12*(-0.22, -0.03) | -0.12 (-0.24, 0.01) | -0.13 (-0.34, 0.09) |
| **Percentage of households that openly defecates in a PSU** | -0.05 (-0.39, 0.28) | -0.06 (-0.29, -0.53) | -0.12 (-0.32, 0.07) | -0.24 (-0.49, 0.01) | -0.04 (-0.47, 0.4) |
| **Place of residence (Ref: Urban)** |  |  |  |  |  |
| Rural | 0.26***(0.08, 0.43) | 0.08 (-0.04, 1.26) | 0.03 (-0.07, 0.14) | -0.04 (-0.17, 0.09) | -0.11 (-0.34, 0.12) |
| **Religion (Ref: Hindus)** |  |  |  |  |  |
| Non-Hindus | 0.26*(0, 0.51) | 0.08 (-0.1, 0.85) | 0.01 (-0.14, 0.16) | -0.02 (-0.21, 0.17) | 0.16 (-0.17, 0.49) |
| **Social class (Ref: SC/ST)** |  |  |  |  |  |
| OBC | 0.17*(0, 0.33) | 0.1 (-0.01, 1.72) | 0.1*(0, 0.2) | 0.02 (-0.1, 0.15) | 0.03 (-0.19, 0.24) |
| Others | 0.22 (-0.41, 0.85) | 0.47*(0.04, 2.12) | 0.39*(0.03, 0.76) | 0.04 (-0.44, 0.51) | -0.09 (-0.91, 0.73) |
| **Wealth Index (Ref: Poor)** |  |  |  |  |  |
| Middle | 0.13 (-0.1, 0.36) | 0.27***(0.12, 3.42) | 0.33***(0.19, 0.46) | 0.32***(0.15, 0.49) | 0.26 (-0.03, 0.56) |
| Rich | 0.13 (-0.1, 0.36) | 0.27***(0.12, 3.42) | 0.33***(0.19, 0.46) | 0.32***(0.15, 0.49) | 0.26 (-0.03, 0.56) |
| **Constant** | -3.75***(-4.4, -3.1) | -2.19***(-2.63, -9.61) | -0.66***(-1.04, -0.28) | 0.8***(0.31, 1.29) | 2.19***(1.35, 3.04) |
