# Supplementary material for: Disparity in childhood stunting in India: Relative importance of community-level nutrition and sanitary practices
Source: PLoS One. 2020 Sep 1;15(9):e0238364. doi: 10.1371/journal.pone.0238364 (PMC7462311; doi:10.1371/journal.pone.0238364)
Supplement: S1 File — (DOCX) [file pone.0238364.s009.docx]

**Supplementary 4**

**Method Description**

**A.1. Re-centered Influence Function (RIF) Decomposition**

**Step 1: Estimating the raw differences in the HAZ scores at the quantiles**

First, we assess the difference in child HAZ score for state A and B (for India time points A and B) by using Kernel Density Estimates. This gives us the raw difference in the HAZ scores at various quantiles. Kernel density estimation (KDE) is a useful statistical tool that helps in smoothing a data set. For the KDE we need to specify a parameter called bandwidth that determines the level of smoothness. In our case, we have defined a bandwidth of 0.10. The estimates are generated by taking the distances of the data points from a point say *H* into consideration. Mathematically, the function, $f_{H}$, for the KDE is expressed as the kernel density function K integrated for all observations in the data,

$\hat{f_{H}}=\int K \left( \frac{H-observation}{bandwidth} \right)$-----------(i)

**Step 2: Deriving the Re-centred Influence Function and regression**

1. **Counterfactual distribution**

In order to go forward with the decomposition, we need to construct a counterfactual distribution. Counterfactual distributions can be computed using simple reweighting procedure. Here, the counterfactual distribution of HAZ specifies what the distribution of HAZ in state A look like if the coefficients were similar to that of state B.

1. **The Influence Function**

The influence function is

IF (HAZ, $Q_{\theta})$= [$\theta-\mathbb{l \{}$HAZ<$Q_{\theta}$}]/$f_{HAZ}(Q_{\theta})$ -------(ii)

Here, $Q_{\theta}$is the $\theta^{th}$ quantile, $\mathbb{l}\left\{ . \right\}$ is an indicator function and $f_{HAZ}$ is the probability density function of the marginal distribution of HAZ at $Q_{\theta}$. The indicator function $\mathbb{l}\left\{ . \right\}$ specifies whether HAZ is greater than or less than $Q_{\theta}$. The sample quantile $Q_{\theta}$ and the density function $f_{HAZ}$ are estimated using kernel density estimates.

1. **Re-centered Influence Function**

The RIF is;

RIF (HAZ, $Q_{\theta}$)= $Q_{\theta}$+ IF (HAZ, $Q_{\theta})$ ------------------(iii)

The transformed variable can be used in an OLS regression for the set of specified covariates. For quantiles, the expected value of RIF regression is an unconditional quantile regression.

The RIF regressions are separately estimated for the different states and the respective counterfactuals. The regression for the $\theta^{th}$ quantile is:

$\hat{RIF}\left( {HAZ}_{K},\hat{Q_{\theta}} \right)= X_{K}\hat{\beta_{K}}$ -------------------(iv)

Here, K represents the states or counterfactuals for which the regression is run.

**Step 3: Decomposition into contribution of individual covariates using Oaxaca-Blinder Decomposition**

In OB decomposition, a linear functional form is assumed as follows:

$Y_{t}={X_{t}^{'}\boldsymbol{ß}}_{t}+ e_{t}$ -----------------(v)

for t = 0,1. Let T define the states between which the HAZ distribution is being compared.

Where, *E*[$e_{t}\left| X_{t}, T=t \right]=E(e_{t})=0$

The difference, $\Delta_{HAZ},$ between the mean HAZ for state A (say T=1) and B (say T=0) is given as:

$\Delta_{HAZ}$=*E*[Y|T=1]- *E*[Y|T=0] -----------------(vi)

The overall mean HAZ gap is to be decomposed into covariate and coefficient effects. Averaging over X, the mean gap can be re-written as:

$\Delta_{HAZ}$=*E*[$Y_{1}]$ –*E*[$Y_{0}]$

=$E[X_{1}\boldsymbol{ß}_{1}+ e_{1}]$ –$E[X_{0}\boldsymbol{ß}_{0}+ e_{0}]$

=$E[X_{1}] \boldsymbol{ß}_{1}$-$E[X_{0}] \boldsymbol{ß}_{0}$ , since $E(e_{t})=0$ and E($\boldsymbol{ß}_{t})=\boldsymbol{ß}_{t}$

=$E[X_{1}] \boldsymbol{ß}_{1}$-$E[X_{1}] \boldsymbol{ß}_{0}+{E[X_{1}] \boldsymbol{ß}}_{0}-E[X_{0}] \boldsymbol{ß}_{0}$ , adding and subtracting the term ${E[X_{1}] \boldsymbol{ß}}_{0}$

=$E[X_{1}] \left( \boldsymbol{ß}_{1}-\boldsymbol{ß}_{0} \right)+\boldsymbol{ß}_{0}\left( E[X_{1}] -E[X_{0}] \right)$

=$\Delta_{HAZ}^{coeff}$+$\Delta_{HAZ}^{cov}$ -------------------------------------------------------------(vii)

Here, ${E[X_{1}] \boldsymbol{ß}}_{0}$is the counterfactual of the distribution of HAZ in state A=1 (the six compared states) if the coefficients were similar to that of state B=0 (Tamil Nadu). For the two time points NFHS 4 is taken as the base group and the counterfactual determines the value of covariates in NFHS 3 if they followed the pattern of association as NFHS 4. The first term, $\Delta_{HAZ}^{coeff}=$ $E[X_{1}]\left( \boldsymbol{ß}_{1}-\boldsymbol{ß}_{0} \right)$ is the coefficient effect and the second term, $\Delta_{HAZ}^{cov}=$ $\boldsymbol{ß}_{0}\left( E[X_{1}] -E[X_{0}] \right)$is the covariate effect. For unconditional quantile regression, the coefficients are estimated and are used for OB decomposition for each quantile.

${\Delta Q_{\theta}}_{HAZ}=E[X_{1_{\theta}}] \left( \boldsymbol{ß}_{1_{\theta}}-\boldsymbol{ß}_{0_{\theta}} \right)+\boldsymbol{ß}_{0_{\theta}}\left( E[X_{1_{\theta}}] -E[X_{0_{\theta}}] \right)$ -----------(viii)

${\Delta Q_{\theta}}_{HAZ}$is defined as the difference between the $\theta^{th}$ quantile estimates of the HAZ distribution between states A and B. $E[X_{t_{\theta}}]$is the mean value of X for place t at the $\theta^{th}$ quantile and $\boldsymbol{ß}_{t_{\theta}}$ is the associated coefficient.

This can be further expanded for different explanatory variables for each quantile as,

${\Delta Q_{\theta}}_{HAZ,k}=E[X_{1_{\theta},k}] \left( \boldsymbol{ß}_{1_{\theta},k}-\boldsymbol{ß}_{0_{\theta},k} \right)+\boldsymbol{ß}_{0_{\theta},k}\left( E[X_{1_{\theta},k}] -E[X_{0_{\theta},k}] \right)$ --------(ix)

where, $X_{t_{\theta},k}$ are the k^th^ independent explanatory variables and $\boldsymbol{ß}_{t_{\theta},k}$ their respective coefficients at the $\theta^{th}$ quantile for the t^th^ place/time. For the sake of simplicity, the error terms have been left out in the equations. However, the model errors are estimated during the statistical analysis. Oaxaca-Blinder procedure is relatively an easy method of decomposing contribution of the explanatory variables from an ordinary least square regression. However, various literature points out that (Barsky, Bound, Charles, & Lupton, 2002; Firpo et al., 2018; Gardeazabal & Ugidos, 2004; R. L. Oaxaca & Ransom, 1999) there are a few disadvantages to this method:

1. The contribution of the variables is sensitive to the choice of the base group.
2. The estimation is consistent only under the linear assumption.
3. Endogeneity is not considered in the model.
